# Supplementary figures and images for: Cytoskeletal vimentin regulates cell size and autophagy through mTORC1 signaling
Source: PLoS Biol. 2022 Sep 13;20(9):e3001737. doi: 10.1371/journal.pbio.3001737 (PMC9469959; doi:10.1371/journal.pbio.3001737)

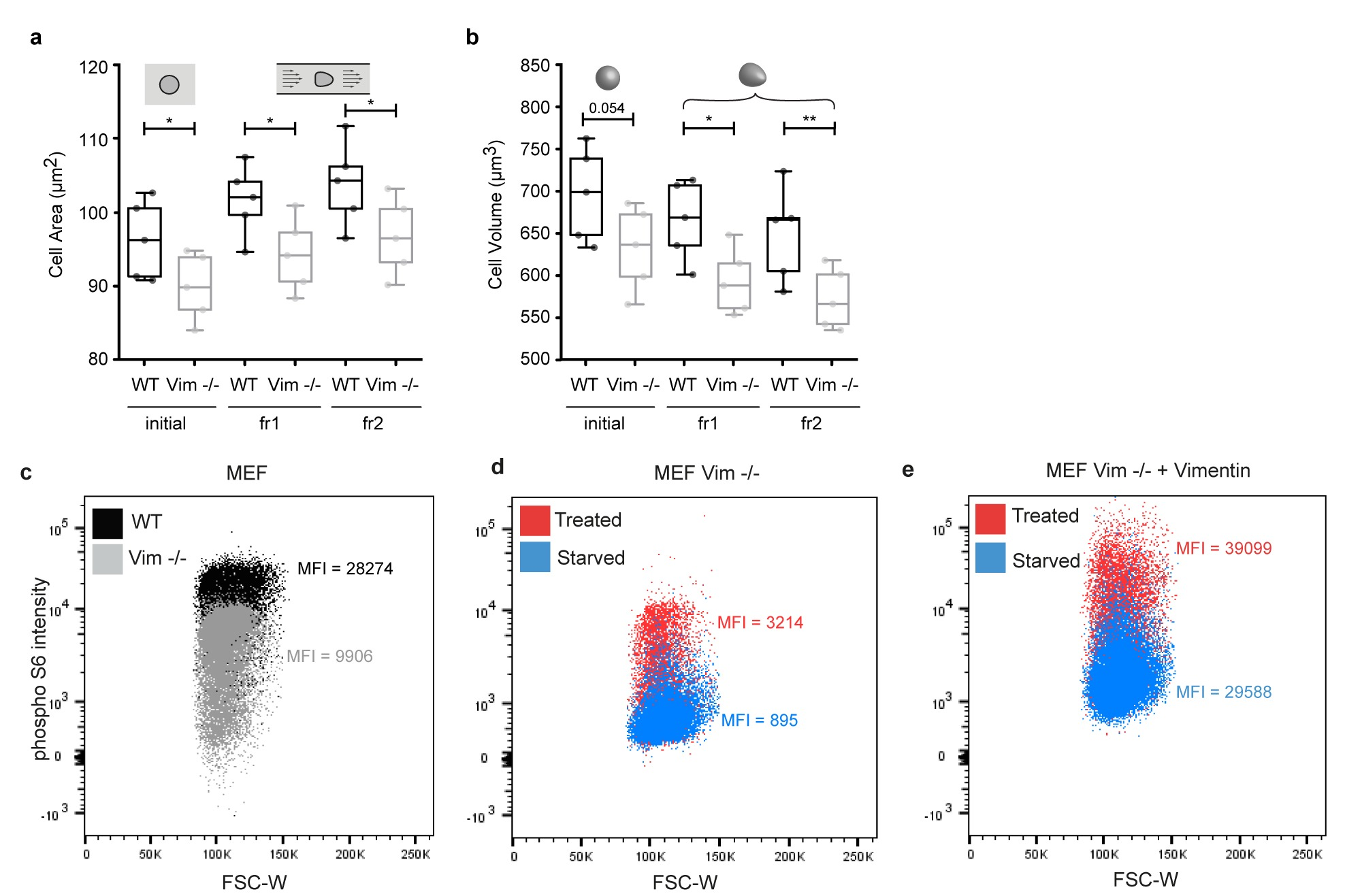

Supplement: S1 Fig — (a) Cell area of WT (black) and Vim −/− (gray) BMDCs obtained from RT-DC measurements of undeformed, spherical cells (initial) and cells deformed in a narrow constriction of a microfluidic channel using 2 different flow rates (fr1 = 0.16 μl/s and fr2 = 0.32 μl/s). (b) Cell volume of WT (black) and Vim −/− (gray) BMDCs corresponding to the samples in (a). In (a) and (b), each data point represents a mean of an independent RT-DC measurement (n = 5), with at least 1,000 cells evaluated per measurement. *p < 0.05, **p < 0.01. RT-DC cell size data has been obtained by reanalyzing recently published measurements (44). (c) Dot plot of phospho-S6 fluorescent intensity in WT (MFI = 28,274) and Vim −/− MEFs (MFI = 9,906). (d) Corresponding dot plot analysis of phospho-S6 fluorescent intensities of Vim −/− MEFs starved (MFI = 895) and nutrient stimulated (MFI = 3,214) compared with (e). Vim −/− MEFs transfected with WT vimentin starved (MFI = 29,588) and nutrient stimulated (MFI = 39,099). Nutrient stimulation was done by starving cells for 1 hour without nutrient and growth factors followed by stimulation with 1× EAAs, 1 mM glutamine, 2.5 mM glucose, and 100 nM insulin for 30 minutes. MFI = median fluorescent intensity of Alexa Fluor 488. One representative of several. Gating used for the analysis can be found in the Figshare https://doi.org/10.6084/m9.figshare.20024534.v1. The data underlying the graphs shown in the S1 Fig can be found in S1 Data. (TIF) [file pbio.3001737.s002.tif]

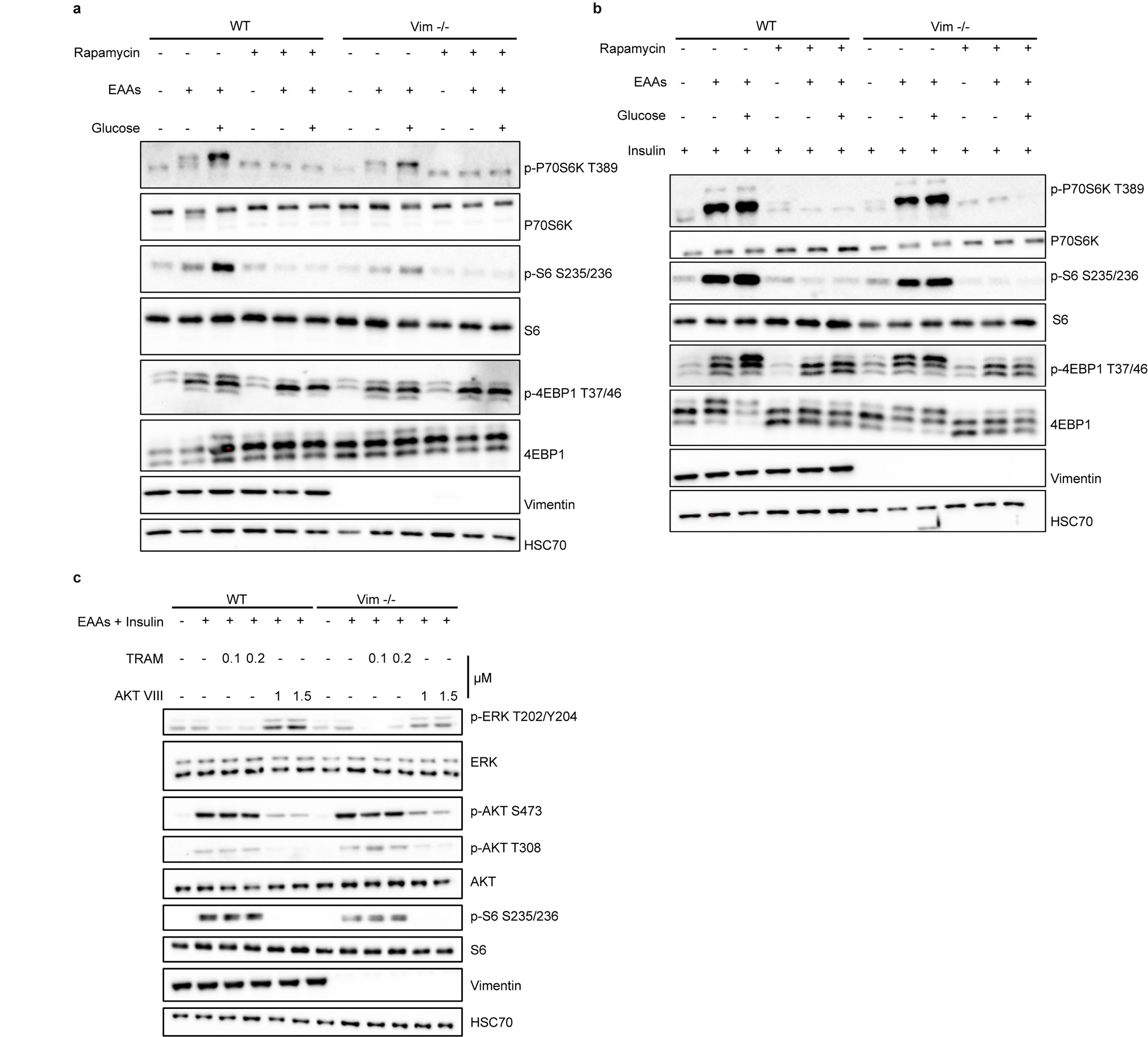

Supplement: S2 Fig — (a) Western blot analysis of WT and Vim −/− MEFs starved with RPMI media lacking amino acids, glucose, and growth factors for 1 hour, followed by stimulation with EAAs or EAAs and NEAAs, with or without 2.5 mM glucose in all combinations. Cells were treated with 100 nM of rapamycin after 40 minutes of starvation (n = 3). (b) Same experiment but including a 100 nM insulin treatment (n = 3). (c) Western blot analysis of WT and Vim −/− MEFs treated as in S2B Fig in the presence of an ERK (TRAM) or an AKT (AKT VIII) inhibitor (n = 3). (TIF) [file pbio.3001737.s003.tif]

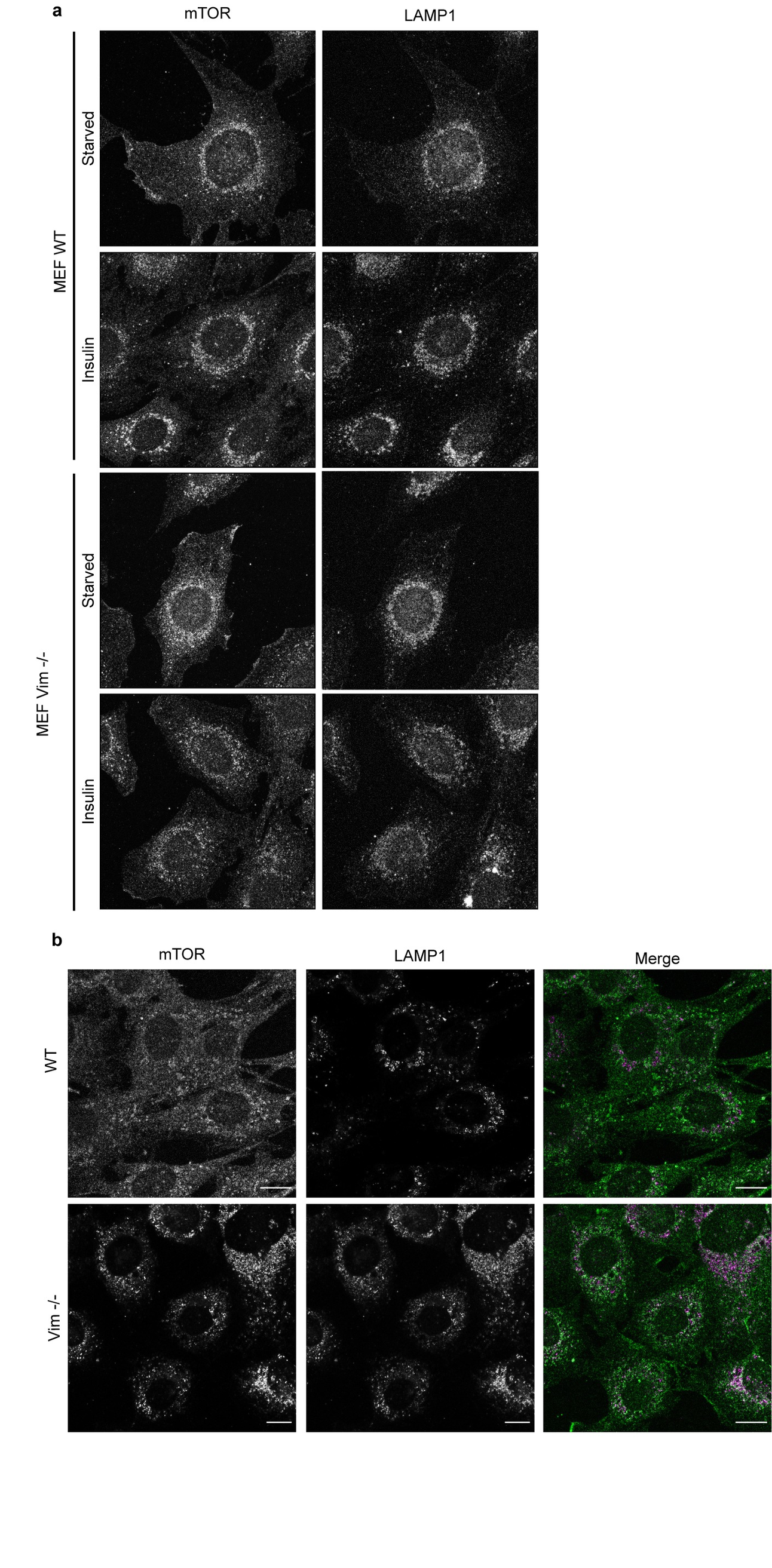

Supplement: S3 Fig — (a) WT and Vim −/− MEFs were serum starved overnight and stimulated with 100 nM insulin for 15 minutes. Cells were stained with mTOR (green channel) and the lysosomal marker LAMP1 (far red channel). (b) Same as in (a), but in steady state conditions (scale bar = 20 μm). (TIF) [file pbio.3001737.s004.tif]

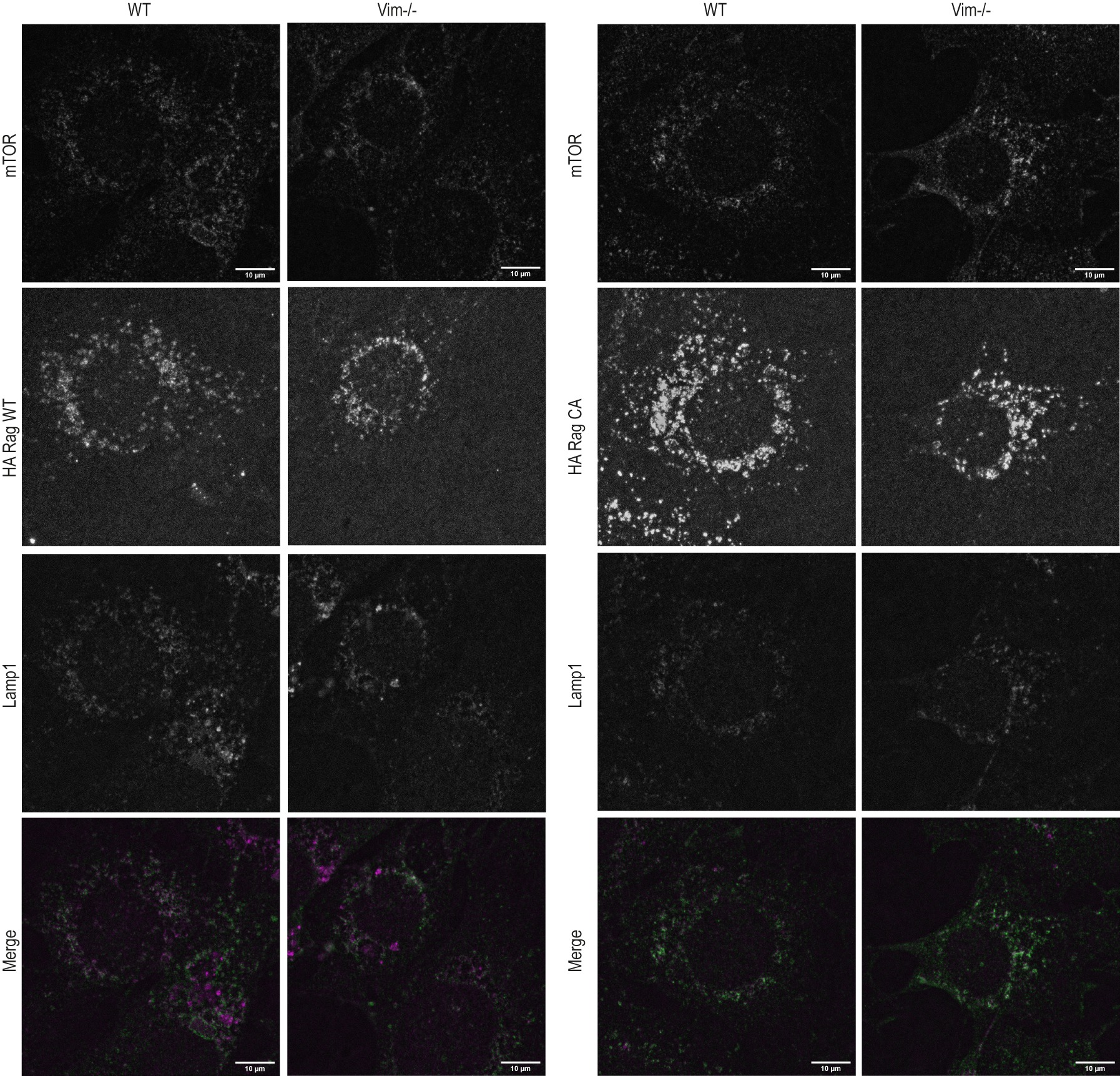

Supplement: S4 Fig — Images show mTOR, HA tagged WT Rag GTPase, or HA tagged constitutively active Rag GTPase and LAMP1 in WT and Vim −/− MEFs. Merged images represent mTOR and LAMP1 channels. (TIF) [file pbio.3001737.s005.tif]

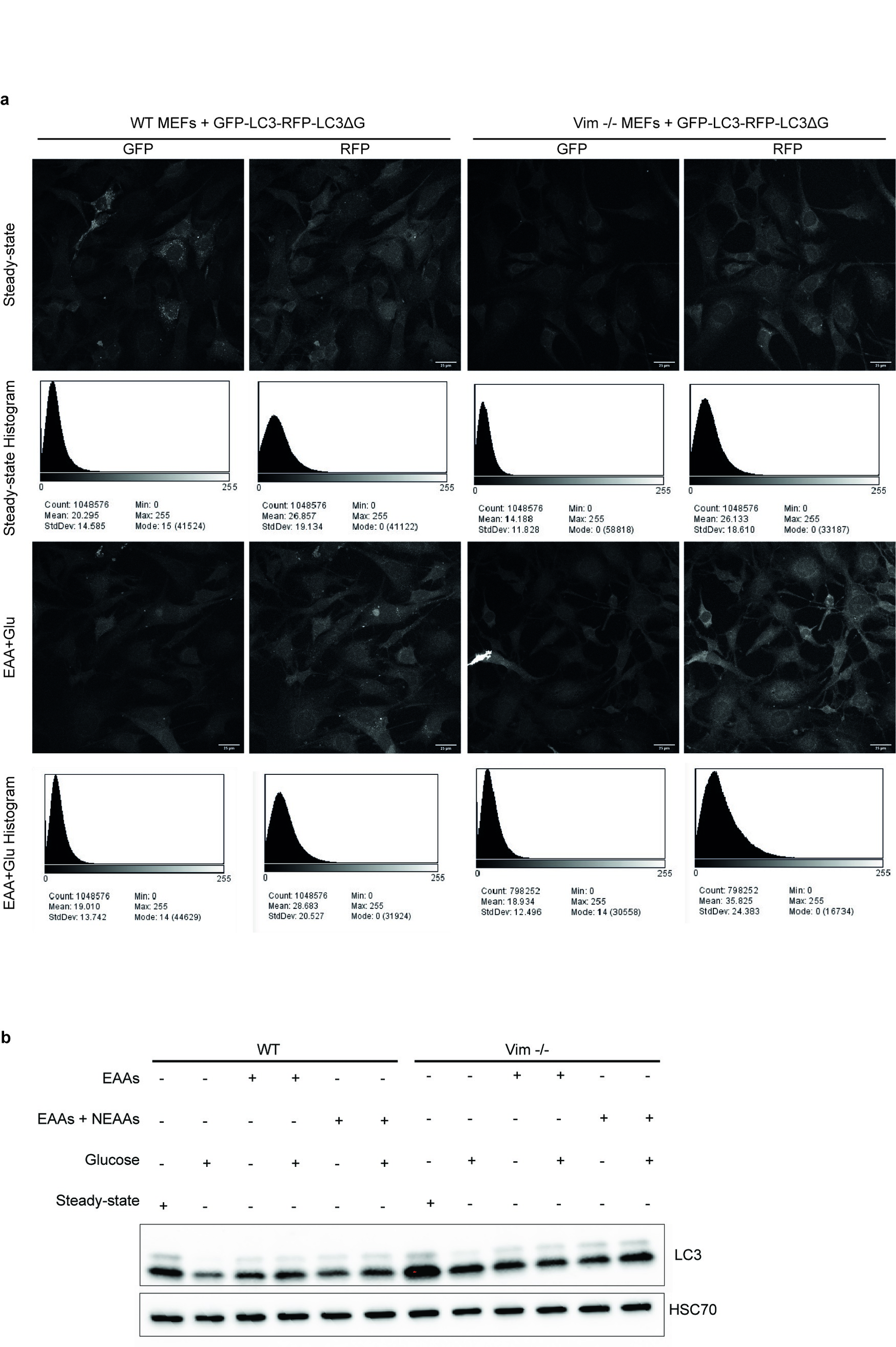

Supplement: S5 Fig — (a) Images show GFP and RFP channels and their intensity histograms of WT and Vim −/− MEFs transfected with pMRX-IP-GFP-LC3-RFP-LC3ΔG. Images were taken under steady-state conditions, serum starvation, and nutrient limitation (only EAAs with L-glutamine and glucose in the media) for 3 hours. (b) Western blot analysis of WT and Vim −/− MEFs grown for 1 hour in normal DMEM with serum or RPMI media lacking amino acids, glucose, and growth factors supplemented with nutrients in different combinations (1 mM L-glutamine plus 1× EAAs alone or with NEAAs and 2.5 mM glucose, n = 3). The data underlying the histogram shown in the S5A Fig can be found in S1 Data. (TIF) [file pbio.3001737.s006.tif]

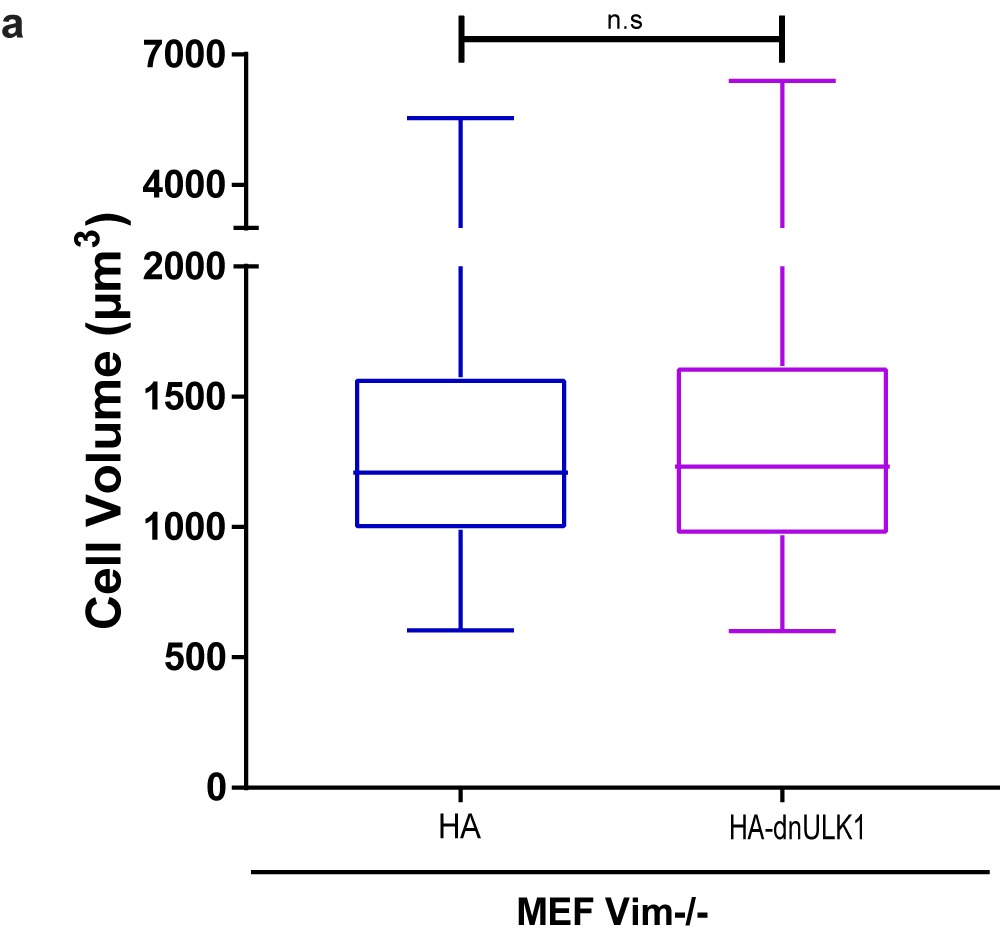

Supplement: S6 Fig — (a) Cell volume distribution of Vim −/− MEFs transfected with HAtag-dnULK1 K46N or HA-tag without insert (n = 3). ns = nonsignificant. The data underlying the graphs shown in the S6 Fig can be found in S1 Data. (TIF) [file pbio.3001737.s007.tif]
