# Supplementary material for: Cytoskeletal vimentin regulates cell size and autophagy through mTORC1 signaling
Source: PLoS Biol. 2022 Sep 13;20(9):e3001737. doi: 10.1371/journal.pbio.3001737 (PMC9469959; doi:10.1371/journal.pbio.3001737)
Supplement: S1 Raw images — (PDF) [file pbio.3001737.s009.pdf]

Fig.2 g

Loading order:
1 - WT MEF
2 - Vim -/- MEF + WT vimentin

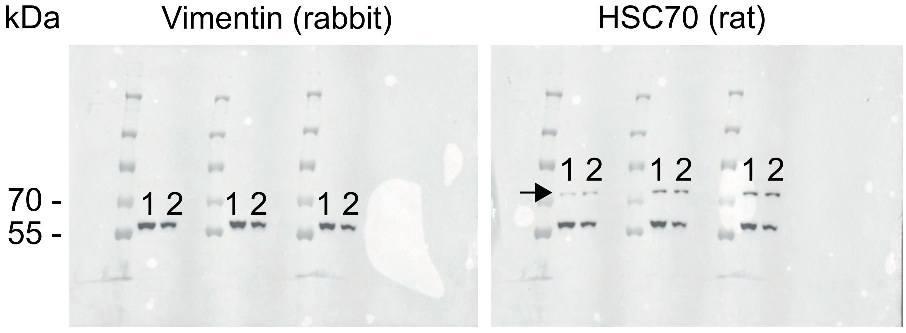

*images are an overlay of the chemiluminescence with the membrane

Fig.3 d

Loading order:
1 - WT MEF serum starved
2 - WT MEF + serum starved +100 nM insulin
3 - WT MEF + serum starved + 5 % serum
4 - Vim -/- MEF serum starved
5 - Vim -/- MEF + serum starved +100 nM insulin
6 - Vim -/- MEF + serum starved + 5 % serum

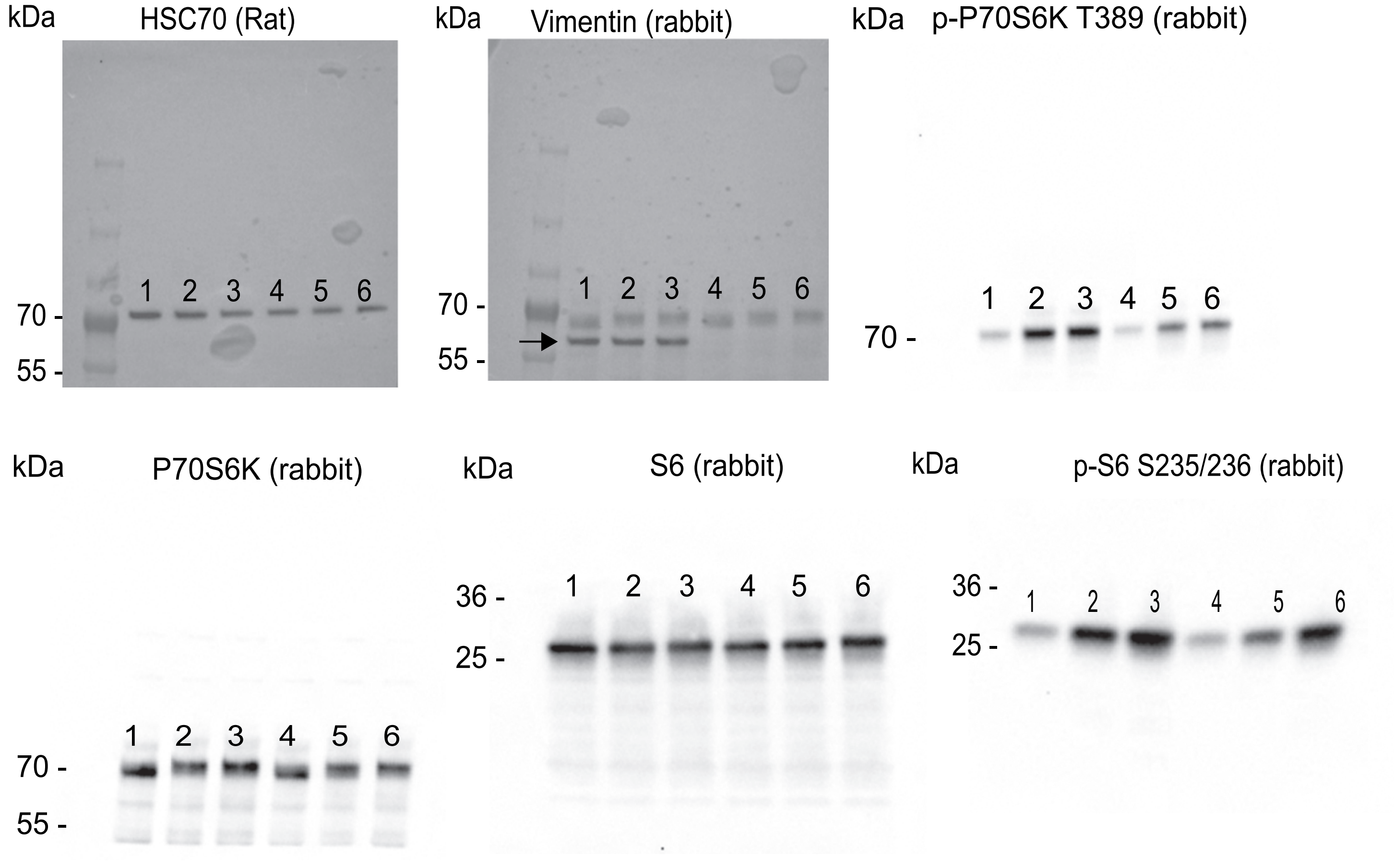

*when possible, images are an overlay of the chemiluminescence with the membrane, otherwise the chemiluminescence alone. Western blot are unedited and uncropped.

Fig.4 a

All samples were starved with RPMI media lacking amino acids, glucose and growth factors for one hour and treated for 30 minutes

Loading order:

1 - WT MEF starved +100 nM insulin
2 - WT MEF starved + 100 nM insulin + 2.5 mM Glucose
3 - WT MEF starved + 100 nM insulin + 1x essential amino acids mix
4 - WT MEF starved + 100 nM insulin + 2.5 mM Glucose + 1x essential amino acids mix
5 - WT MEF starved +100 nM insulin + 1x non-essential and essential amino acids mix
6 - WT MEF starved +100 nM insulin + 2.5 mM Glucose + 1x non-essential and essential amino acids mix
7 - Vim -/- MEF starved +100 nM insulin
8 - Vim -/- MEF starved + 100 nM insulin + 2.5 mM Glucose
9 - Vim -/- MEF starved + 100 nM insulin + 1x essential amino acids mix
10 - Vim -/- MEF starved + 100 nM insulin + 2.5 mM Glucose + 1x essential amino acids mix
11 - Vim -/- MEF starved +100 nM insulin + 1x non-essential and essential amino acids mix
12-Vim -/- MEF starved +100 nM insulin + 2.5 mM Glucose + 1x non-essential and essential amino acids mix

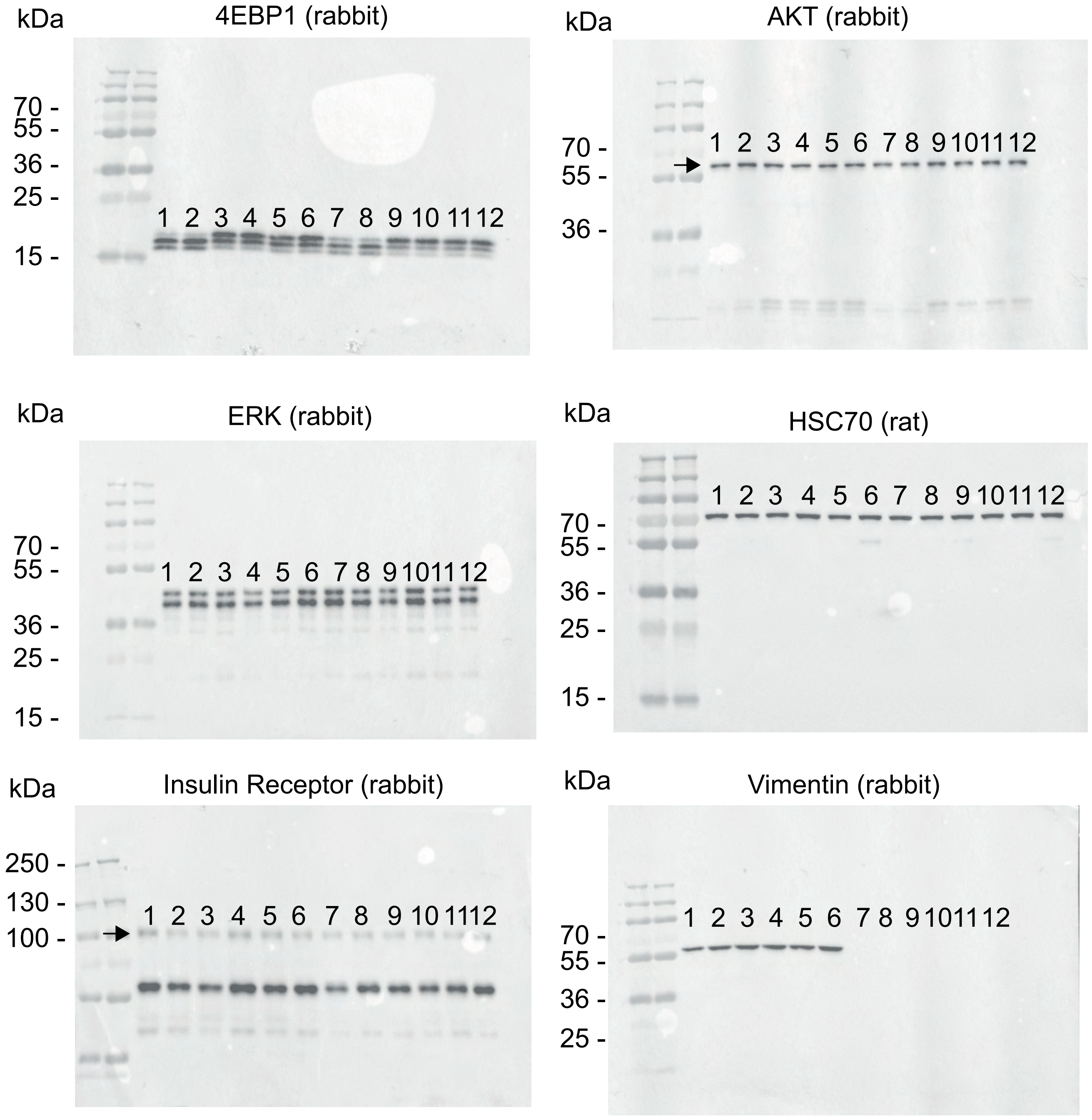

*when possible, images are an overlay of the chemiluminescence with the membrane, otherwise the chemiluminescence alone. Western blot are unedited and uncropped.

Fig.4 a continued

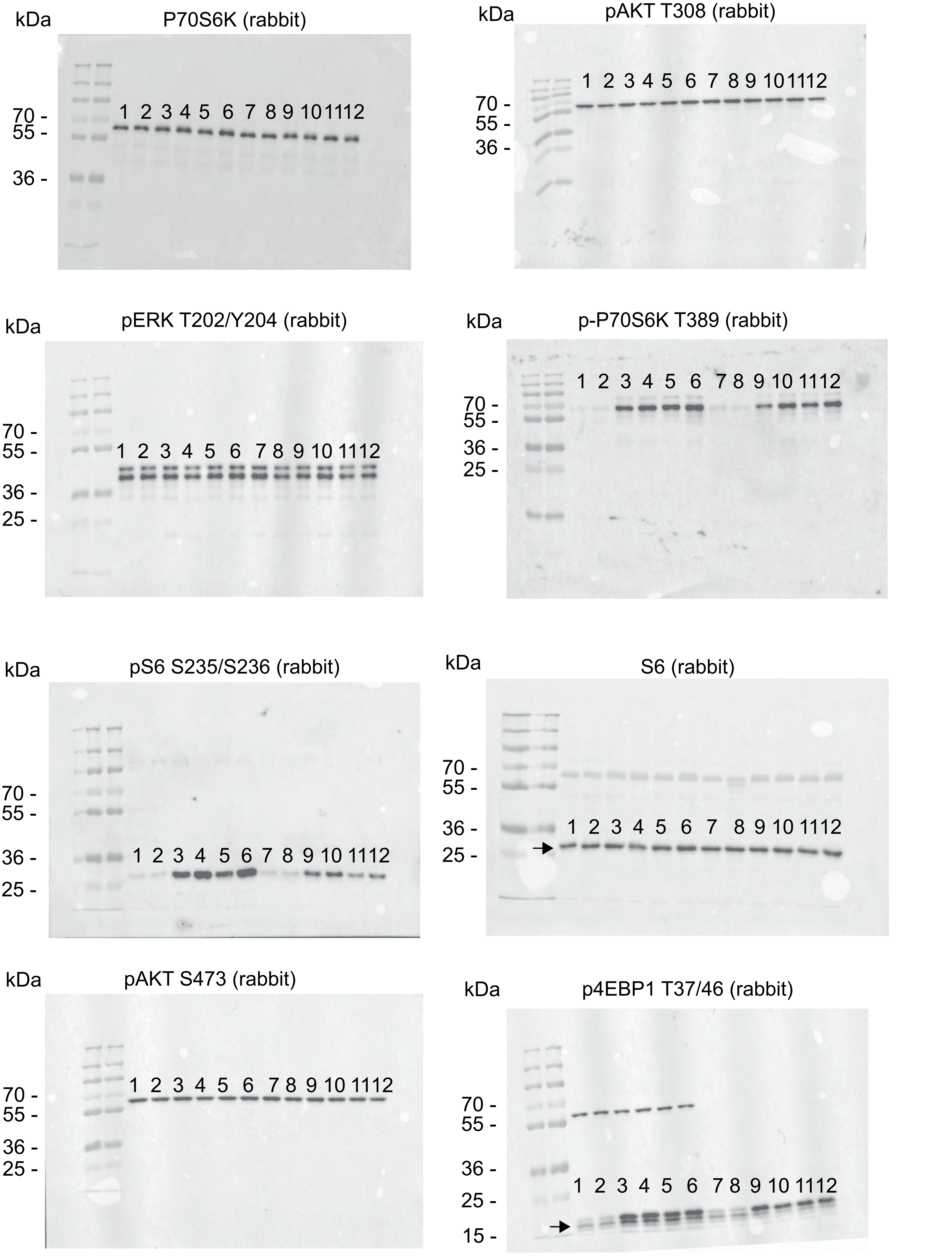

*when possible, images are an overlay of the chemiluminescence with the membrane, otherwise the chemiluminescence alone. Western blot are unedited and uncropped.

Fig.4 a continued

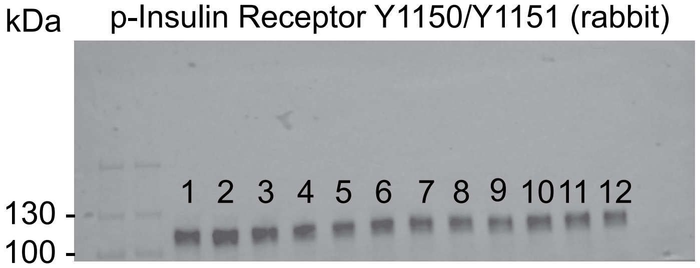

Fig.4 b

All samples were starved with RPMI media lacking amino acids, glucose and growth factors for one hour and treated for 30 minutes

Loading order:

1 - WT MEF starved

2 - WT MEF starved + 2.5 mM Glucose

3 - WT MEF starved + 1x essential amino acids mix

4 - WT MEF starved + 2.5 mM Glucose + 1x essential amino acids mix

5 - WT MEF starved + 1x non-essential and essential amino acids mix

6 - WT MEF starved + 2.5 mM Glucose + 1x non-essential and essential amino acids mix

7 - Vim -/- MEF starved

8 - Vim -/- MEF starved + 2.5 mM Glucose

9 - Vim -/- MEF starved + 1x essential amino acids mix

10 - Vim -/- MEF starved + 2.5 mM Glucose + 1x essential amino acids mix

11 - Vim -/- MEF starved + 1x non-essential and essential amino acids mix

12 - Vim -/- MEF starved + 2.5 mM Glucose + 1x non-essential and essential amino acids mix

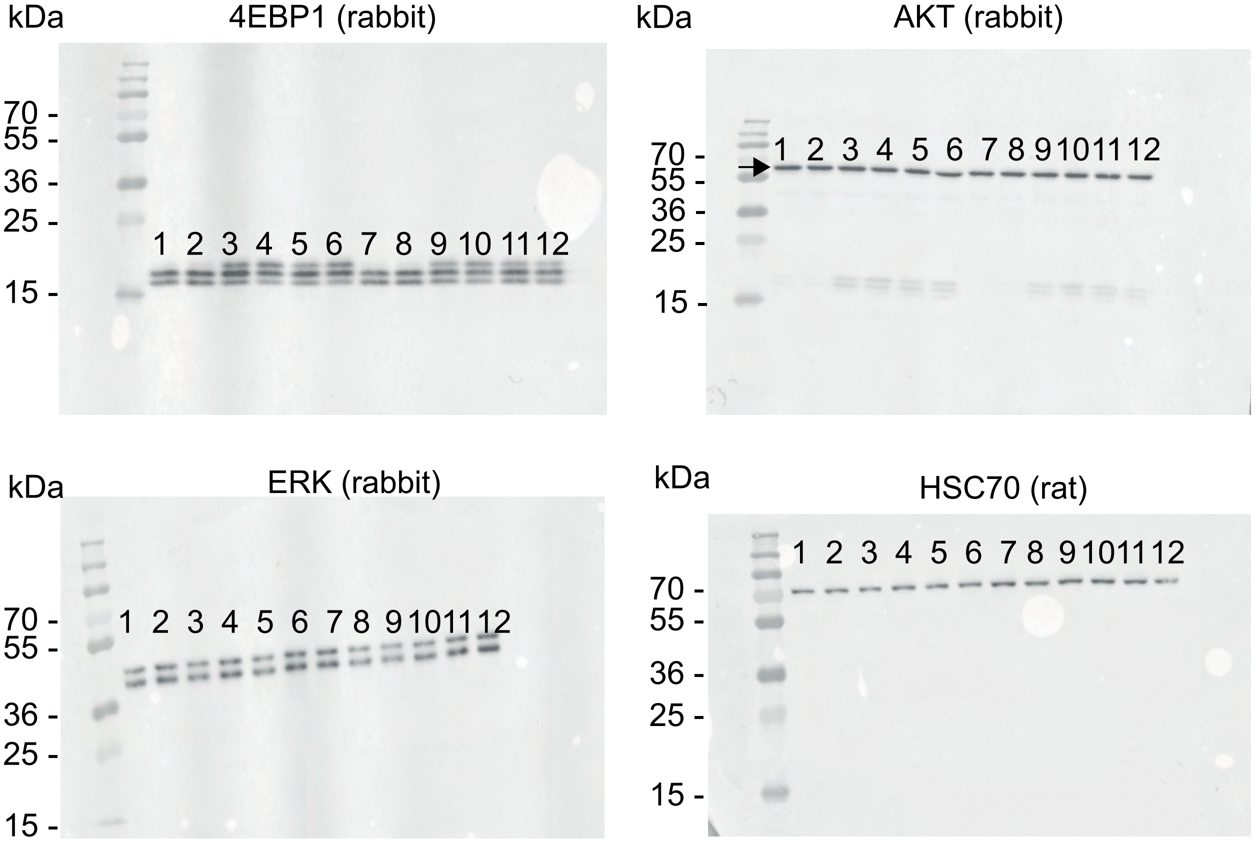

*when possible, images are an overlay of the chemiluminescence with the membrane, otherwise the chemiluminescence alone. Western blot are unedited and uncropped.

Fig.4 b continued

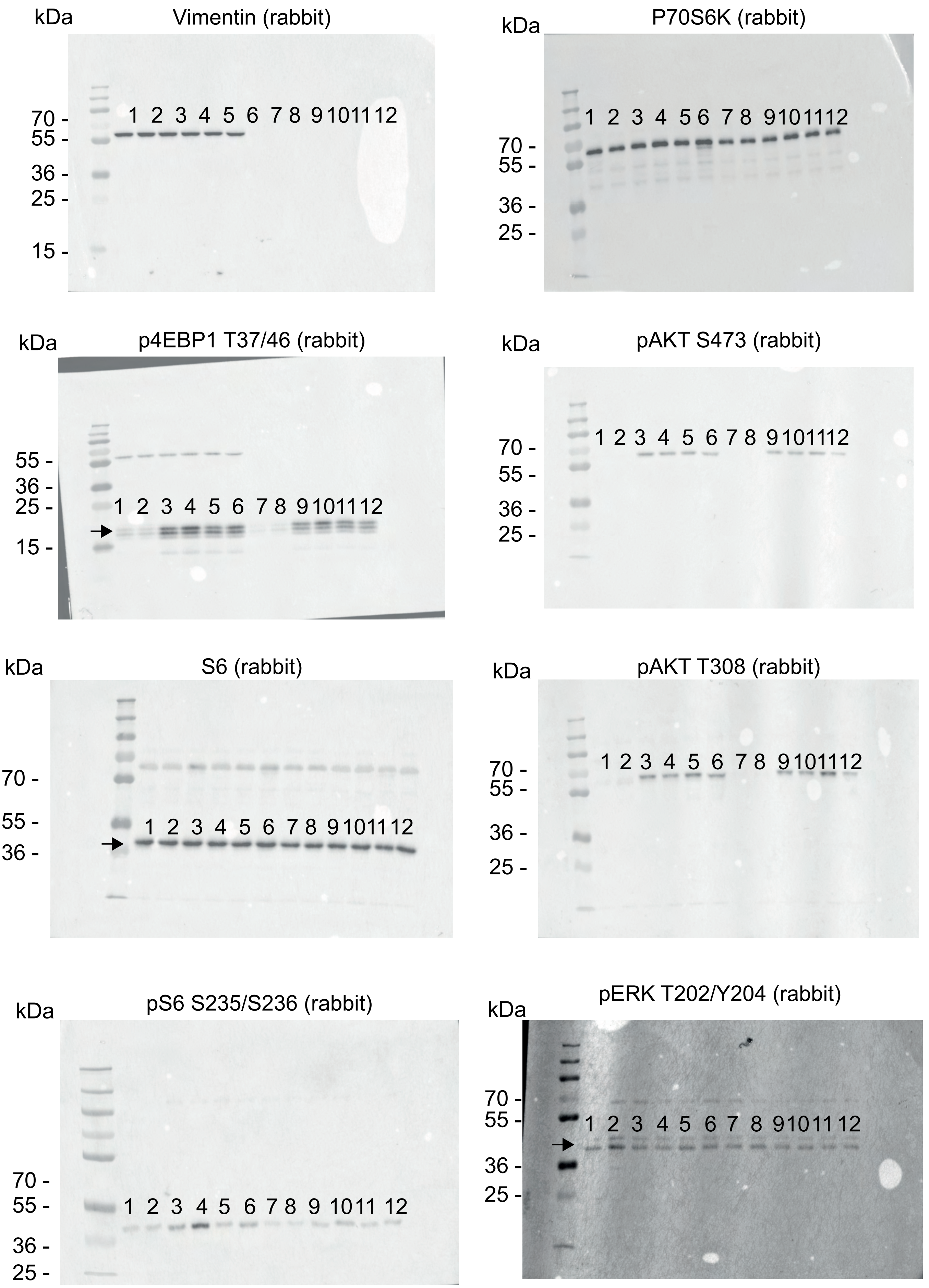

*when possible, images are an overlay of the chemiluminescence with the membrane, otherwise the chemiluminescence alone. Western blot are unedited and uncropped.

Fig.4 b continued

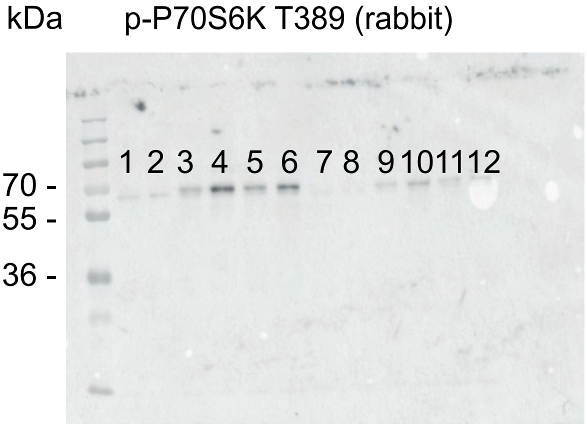

Fig.6 a

All samples were starved with RPMI media lacking amino acids, glucose and growth factors for one hour and treated for 30 minutes

Loading order:

1 - WT MEF starved +100 nM insulin
2 - WT MEF starved + 100 nM insulin + 2.5 mM Glucose
3 - WT MEF starved + 100 nM insulin + 1x essential amino acids mix
4 - WT MEF starved + 100 nM insulin + 2.5 mM Glucose + 1x essential amino acids mix
5 - WT MEF starved +100 nM insulin + 1x non-essential and essential amino acids mix
6 - WT MEF starved +100 nM insulin + 2.5 mM Glucose + 1x non-essential and essential amino acids mix
7 - Vim -/- MEF starved +100 nM insulin
8 - Vim -/- MEF starved + 100 nM insulin + 2.5 mM Glucose
9 - Vim -/- MEF starved + 100 nM insulin + 1x essential amino acids mix
10 - Vim -/- MEF starved + 100 nM insulin + 2.5 mM Glucose + 1x essential amino acids mix
11 - Vim -/- MEF starved +100 nM insulin + 1x non-essential and essential amino acids mix
12-Vim -/- MEF starved +100 nM insulin + 2.5 mM Glucose + 1x non-essential and essential amino acids mix

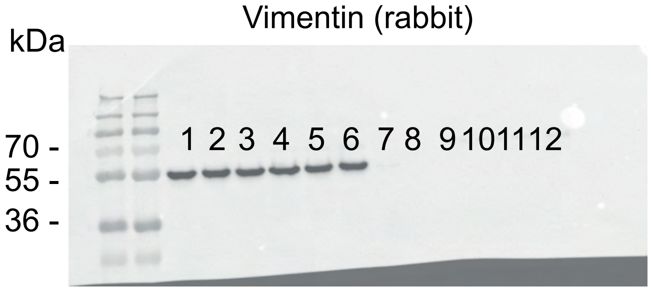

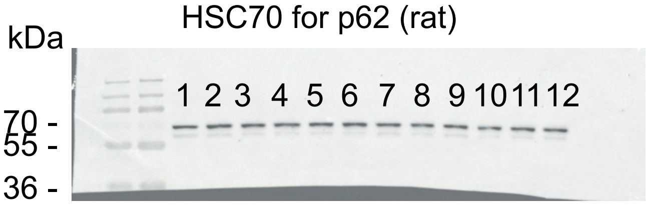

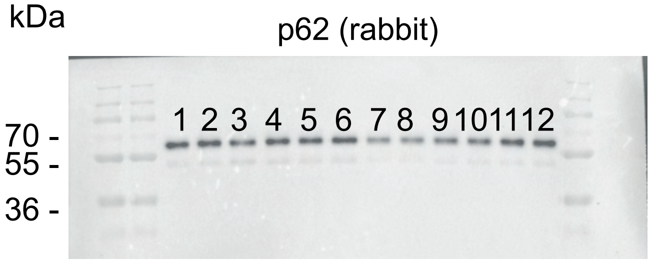

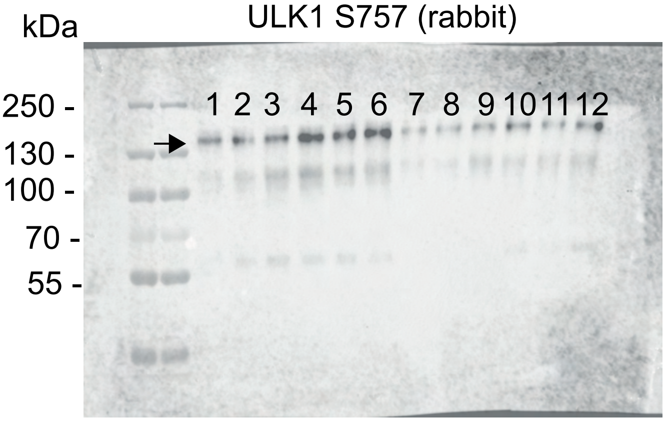

*when possible, images are an overlay of the chemiluminescence with the membrane, otherwise the chemiluminescence alone. Western blot are unedited and uncropped.

Fig.6 a continued

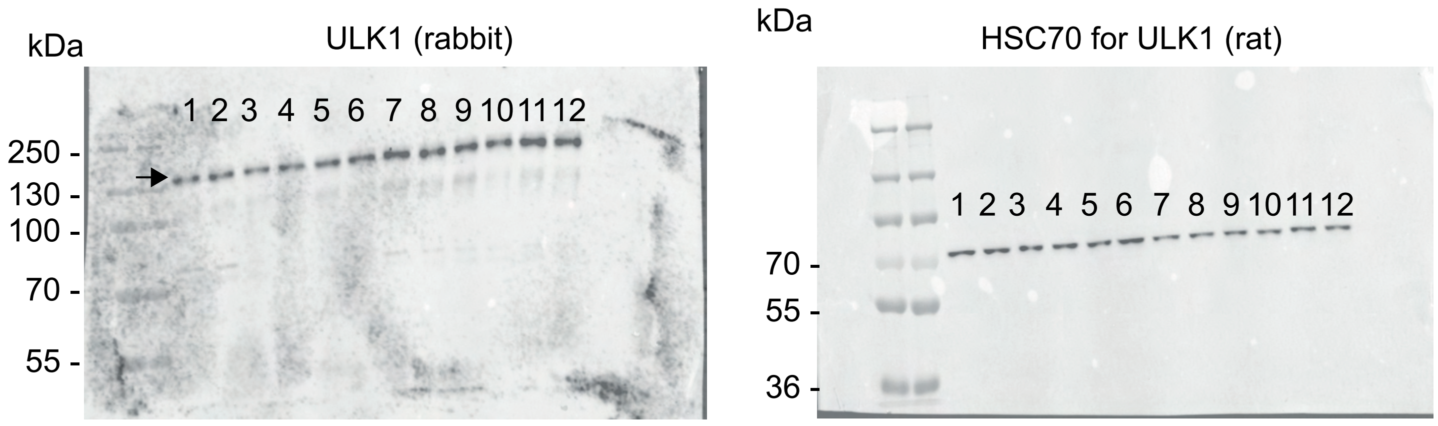

Fig.6 b

All samples were maintained for one hour in RPMI media with different compositions

Loading order:

1 - WT MEF in starvation RPMI
2 - WT MEF in starvation RPMI with 2.5 mM Glucose
3 - WT MEF in starvation RPMI with 1x essential amino acids mix
4 - WT MEF in starvation RPMI with 2.5 mM Glucose and 1x essential amino acids mix
5 - WT MEF in starvation RPMI with 1x non-essential amino acids mix
6 - WT MEF in starvation RPMI with 2.5 mM Glucose and 1x non-essential amino acids mix
7 - Vim -/- MEF in starvation RPMI
8 - Vim -/- MEF in starvation RPMI with 2.5 mM Glucose
9 - Vim -/- MEF in starvation RPMI with 1x essential amino acids mix
10 - Vim -/- MEF in starvation RPMI with 2.5 mM Glucose and 1x essential amino acids mix
11 - Vim -/- MEF in starvation RPMI with 1x non-essential amino acids mix
12 - Vim -/- MEF in starvation RPMI with 2.5 mM Glucose and 1x non-essential amino acids mix

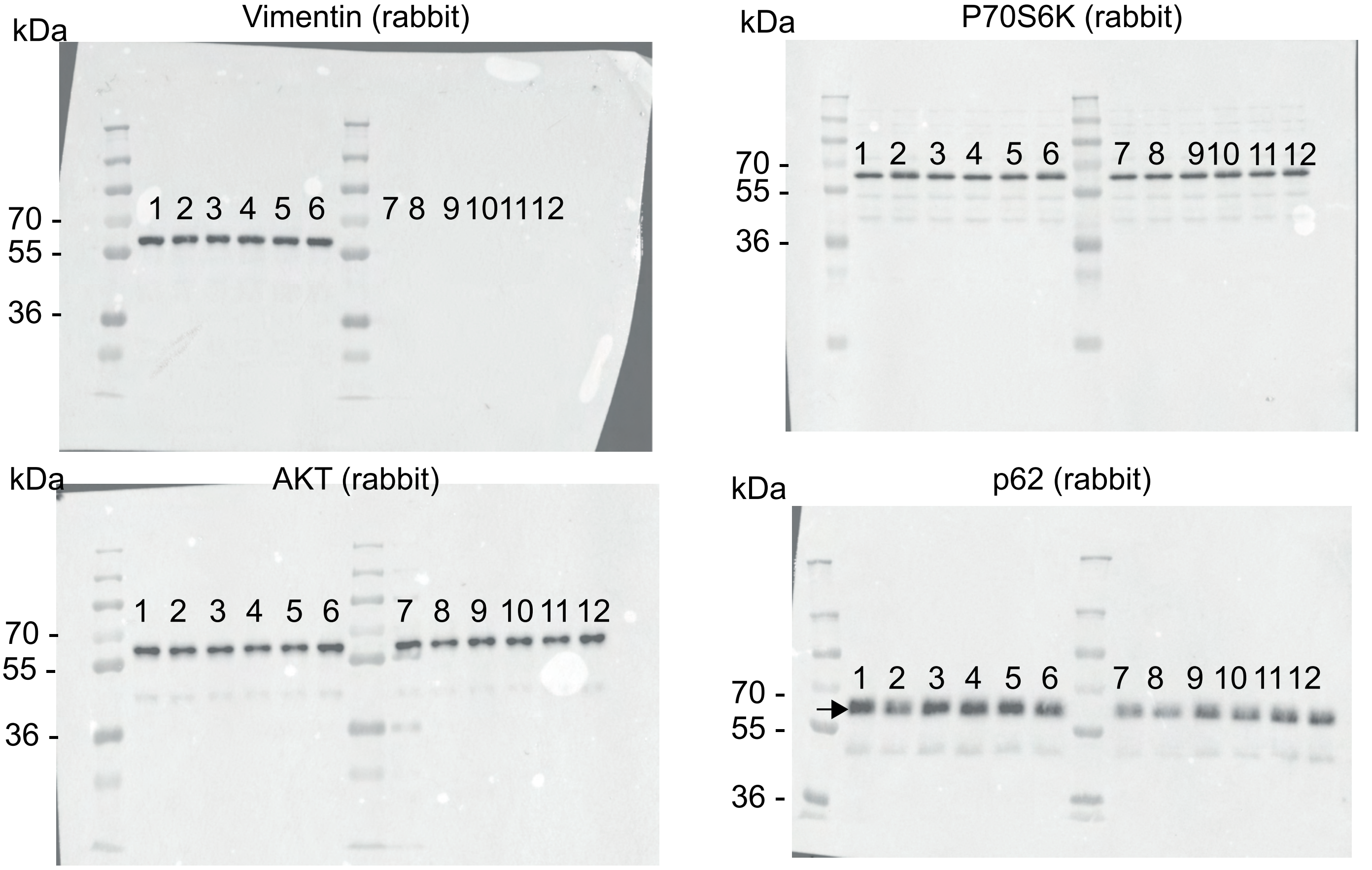

*when possible, images are an overlay of the chemiluminescence with the membrane, otherwise the chemiluminescence alone. Western blot are unedited and uncropped.

Fig.6 b continued

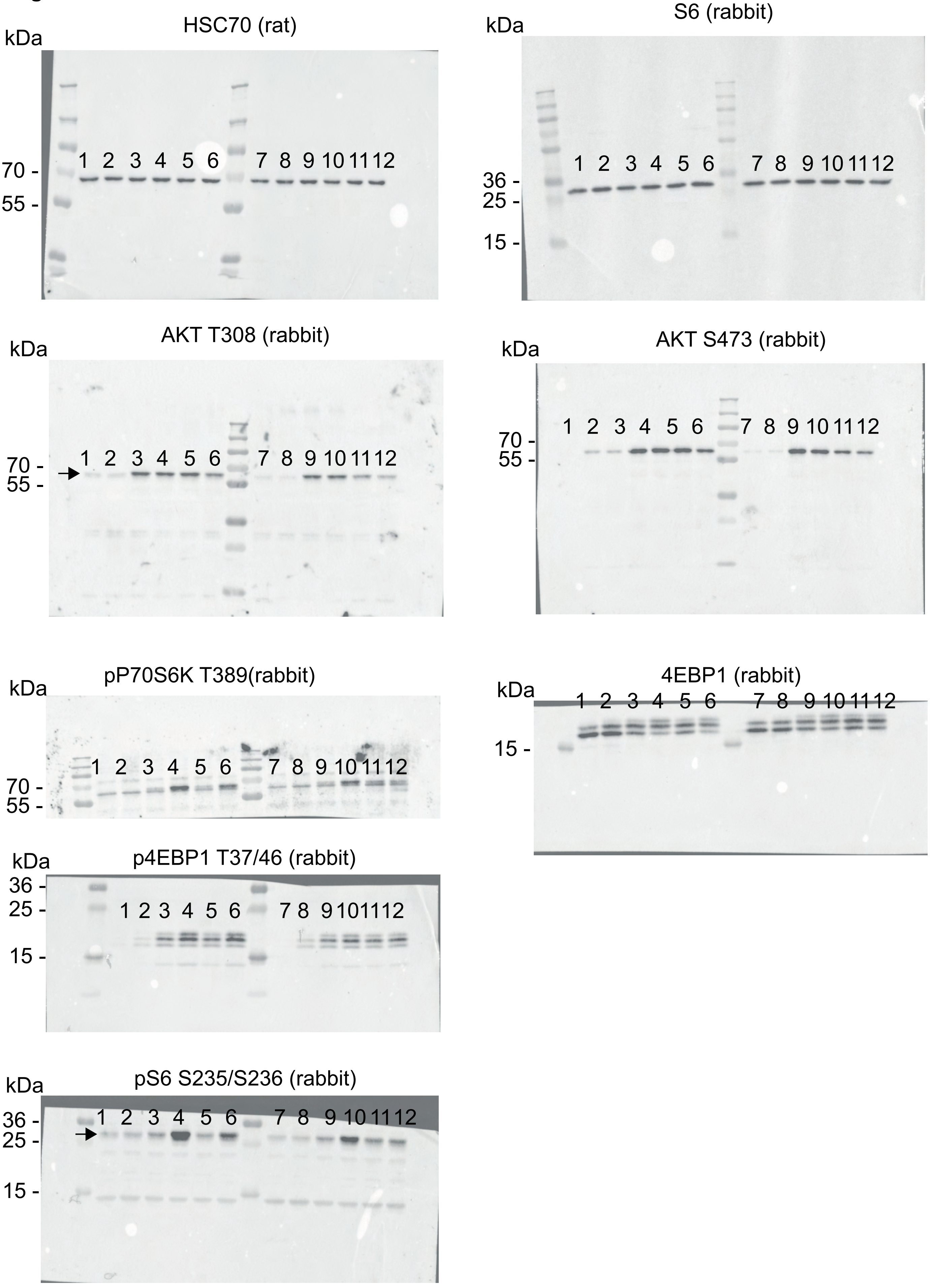

*when possible, images are an overlay of the chemiluminescence with the membrane, otherwise the chemiluminescence alone. Western blot are unedited and uncropped.

Fig.6 d

Loading order:
1 - WT MEF in steaty state
2 - WT MEF in starvation RPMI with 2.5 mM Glucose
3 - WT MEF in starvation RPMI with 1x essential amino acids
4 - WT MEF in starvation RPMI with 2.5 mM Glucose and 1x essential amino acids
5 - WT MEF in starvation RPMI with 1x non-essential amino acids
6 - WT MEF in starvation RPMI with 2.5 mM Glucose and 1x non-essential amino acids
7 - Vim -/- MEF in steady state
8 - Vim -/- MEF in starvation RPMI with 2.5 mM Glucose
9 - Vim -/- MEF in starvation RPMI with 1x essential amino acids
10 - Vim -/- MEF in starvation RPMI with 2.5 mM Glucose and 1x essential amino acids
11 - Vim -/- MEF in starvation RPMI with 1x non-essential amino acids
12 - Vim -/- MEF in starvation RPMI with 2.5 mM Glucose and 1x non-essential amino acids

Vimentin (rabbit)

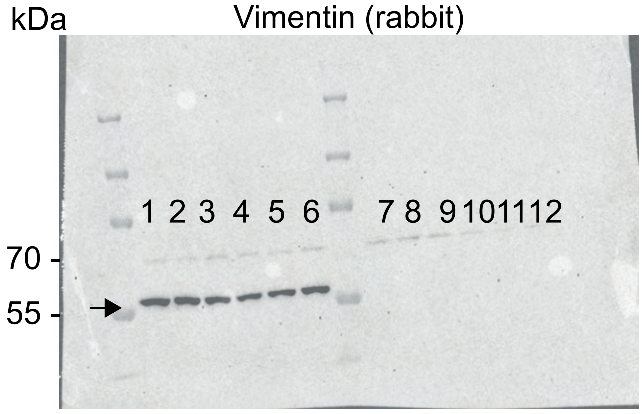

ULK1 (rabbit)

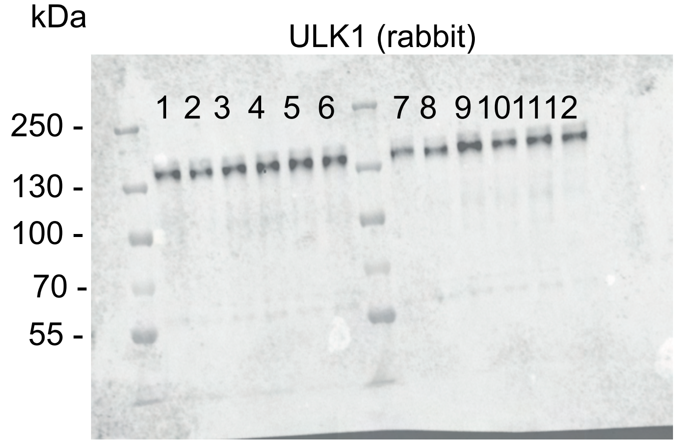

HSC70 (rat)

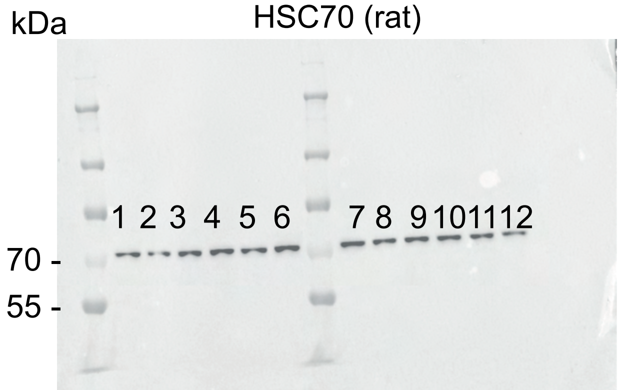

ULK1 S757 (rabbit)

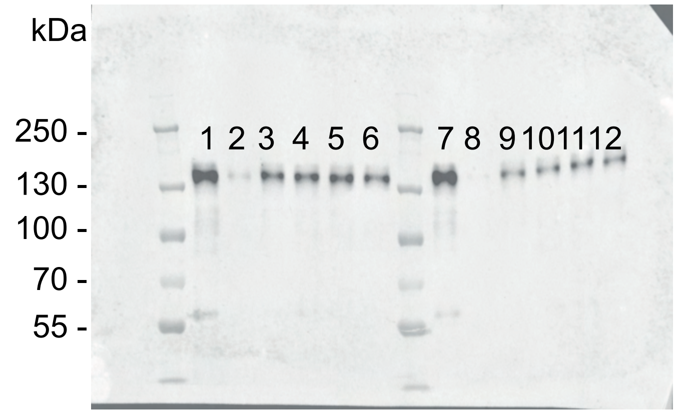

*when possible, images are an overlay of the chemiluminescence with the membrane, otherwise the chemiluminescence alone. Western blot are unedited and uncropped.

Fig.S2 a

All samples were starved with RPMI media lacking amino acids, glucose and growth factors for one hour and treated for 30 minutes. Rapamycin was added after 40 minutes of starvation.
Loading order:
1 - WT MEF starved
2 - WT MEF starved + 1x non-essential and essential amino acids mix
3 - WT MEF starved + 2.5 mM Glucose + 1x non-essential and essential amino acids mix
4 - WT MEF starved + 100 nM rapamycin
5 - WT MEF starved + 1x non-essential and essential amino acids mix + 100 nM rapamycin
6 - WT MEF starved + 2.5 mM Glucose + 1x non-essential and essential amino acids mix + 100 nM rapamycin
7 - Vim -/- MEF starved
8 - Vim -/- MEF starved + 1x non-essential and essential amino acids mix
9 - Vim -/- MEF starved + 2.5 mM Glucose + 1x non-essential and essential amino acids mix
10 - Vim -/- MEF starved + 100 nM rapamycin
11 - Vim -/- MEF starved + 1x non-essential and essential amino acids mix + 100 nM rapamycin
12 - Vim -/- MEF starved + 2.5 mM Glucose + 1x non-essential and essential amino acids mix + 100 nM rapamycin

HSC70 (rat)

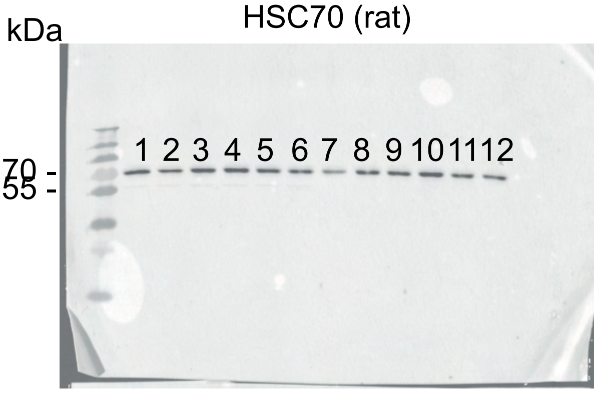

pS6 S235/S236 (rabbit)

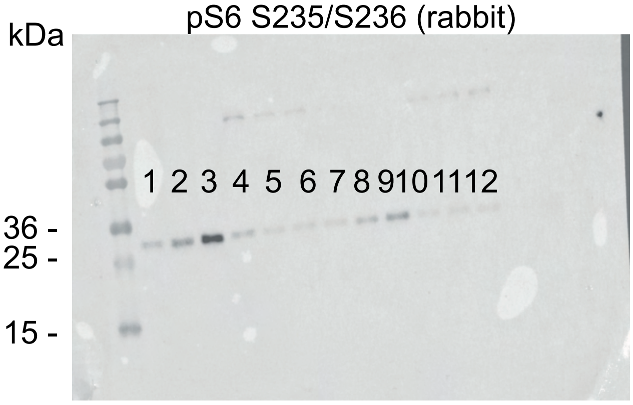

Vimentin (rabbit)

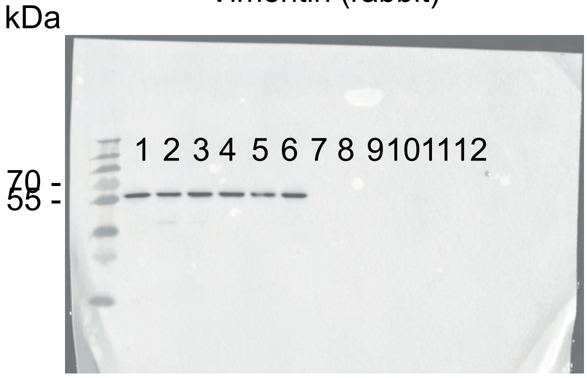

S6 (rabbit)

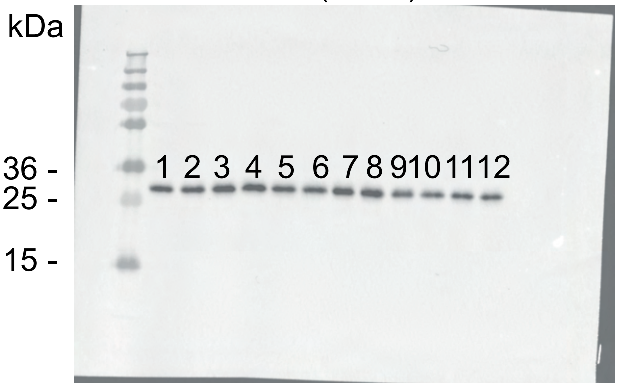

*when possible, images are an overlay of the chemiluminescence with the membrane, otherwise the chemiluminescence alone. Western blot are unedited and uncropped.

Fig.S2 a continued

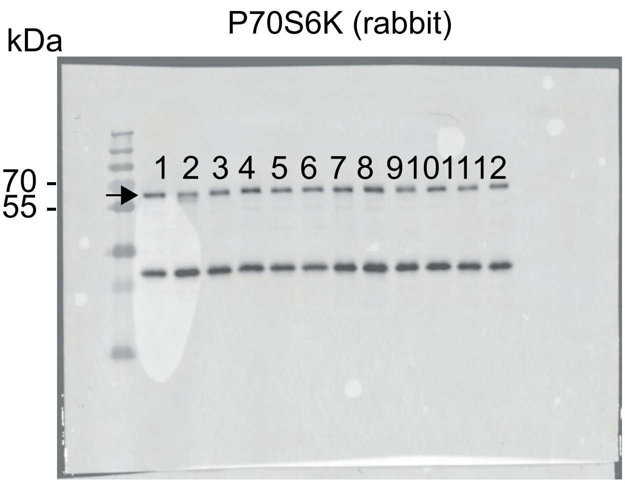

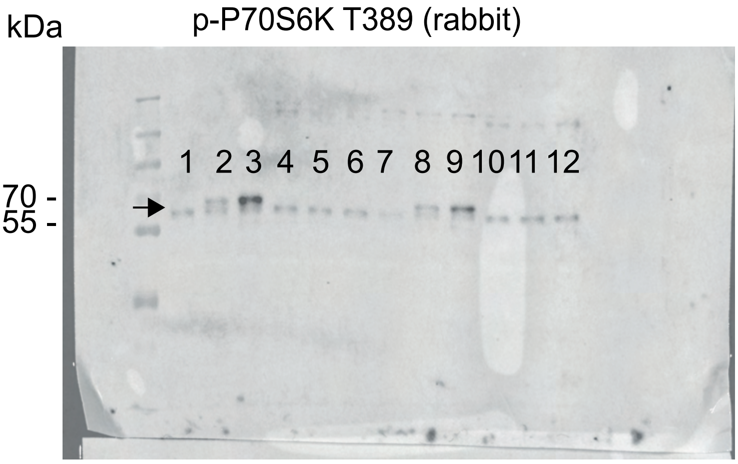

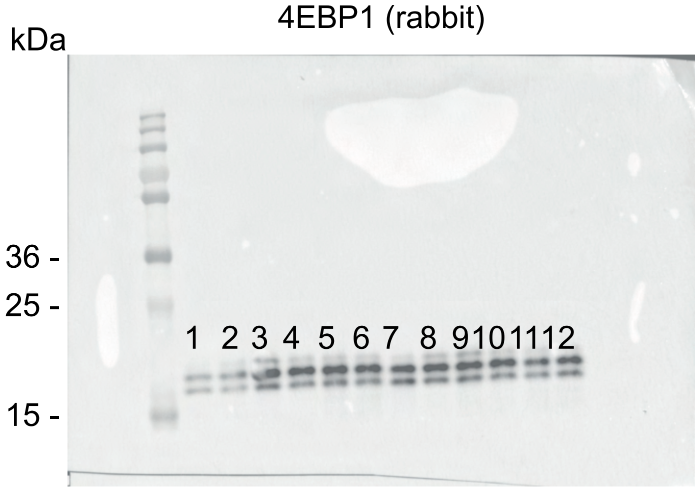

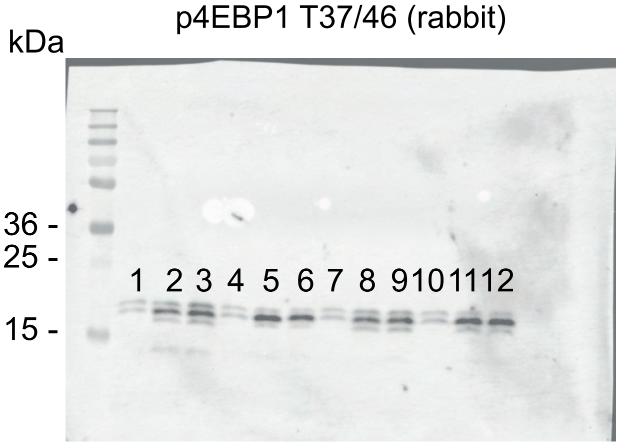

*when possible, images are an overlay of the chemiluminescence with the membrane, otherwise the chemiluminescence alone. Western blot are unedited and uncropped.

Fig.S2 b

All samples were starved with RPMI media lacking amino acids, glucose and growth factors for one hour and treated for 30 minutes. Rapamycin was added after 40 minutes of starvation.

Loading order:

1 - WT MEF starved +100 nM insulin
2 - WT MEF starved +100 nM insulin + 1x non-essential and essential amino acids mix
3 - WT MEF starved +100 nM insulin + 2.5 mM Glucose + 1x non-essential and essential amino acids mix
4 - WT MEF starved +100 nM insulin + 100 nM rapamycin
5 - WT MEF starved +100 nM insulin + 1x non-essential and essential amino acids mix + 100 nM rapamycin
6 - WT MEF starved +100 nM insulin + 2.5 mM Glucose + 1x non-essential and essential amino acids mix + 100 nM rapamycin
7 - Vim -/- MEF starved +100 nM insulin
8 - Vim -/- MEF starved +100 nM insulin + 1x non-essential and essential amino acids mix
9 - Vim -/- MEF starved +100 nM insulin + 2.5 mM Glucose + 1x non-essential and essential amino acids mix
10 - Vim -/- MEF starved +100 nM insulin + 100 nM rapamycin
11 - Vim -/- MEF starved +100 nM insulin + 1x non-essential and essential amino acids mix + 100 nM rapamycin
12 - Vim -/- MEF starved +100 nM insulin + 2.5 mM Glucose + 1x non-essential and essential amino acids + 100 nM rapamycin

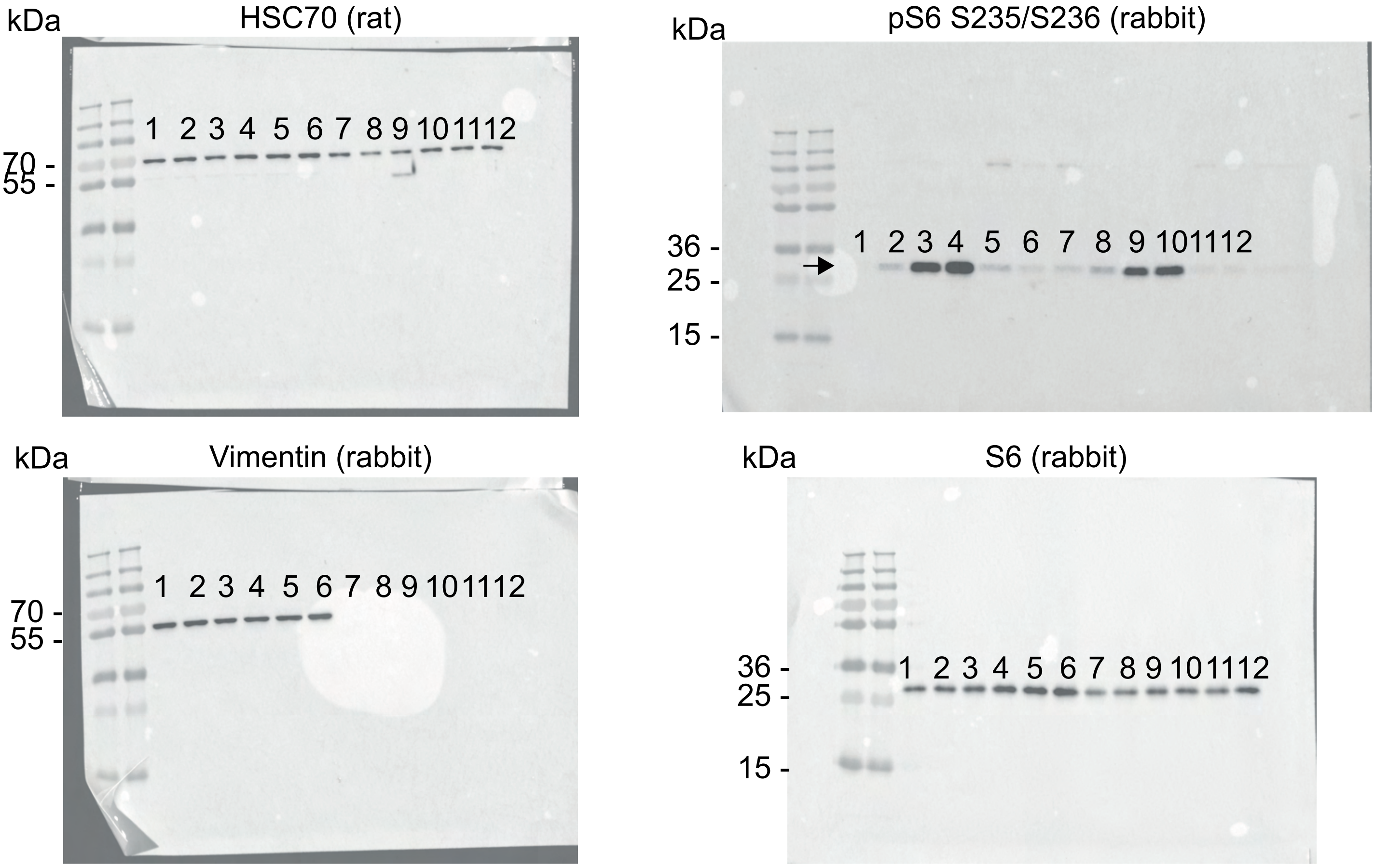

*when possible, images are an overlay of the chemiluminescence with the membrane, otherwise the chemiluminescence alone. Western blot are unedited and uncropped.

Fig.S2 b continued

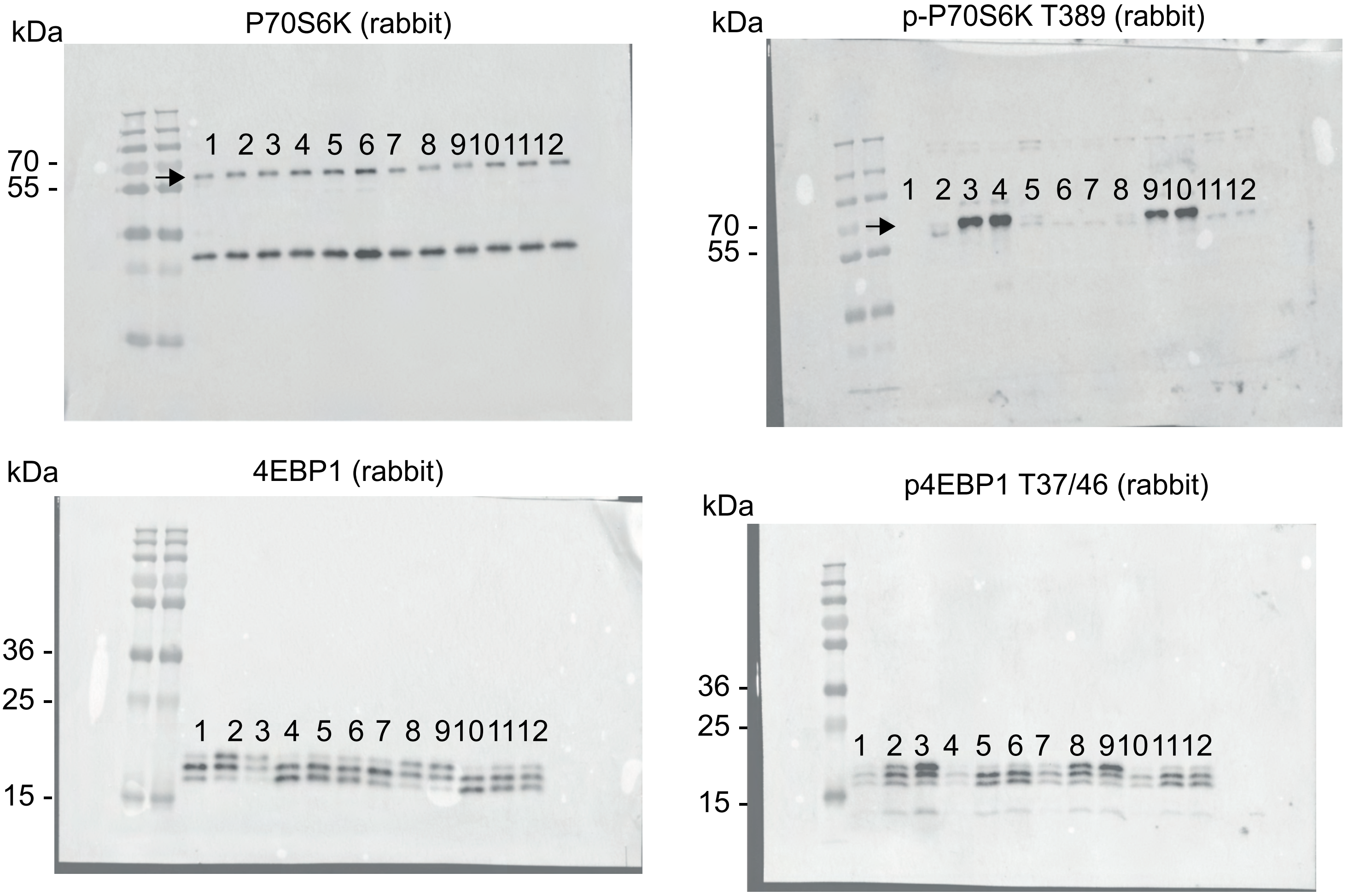

*when possible, images are an overlay of the chemiluminescence with the membrane, otherwise the chemiluminescence alone. Western blot are unedited and uncropped.

Fig.S2 c

All samples were starved with RPMI media lacking amino acids, glucose and growth factors for one hour and treated for 30 minutes

Loading order:

1 - WT MEF starved
2 - WT MEF starved +100 nM insulin + 2.5 mM Glucose + 1x non-essential and essential amino acids mix
3 - WT MEF starved +100 nM insulin + 2.5 mM Glucose + 1x non-essential and essential amino acids mix + 0,1 µM TRAM
4 - WT MEF starved +100 nM insulin + 2.5 mM Glucose + 1x non-essential and essential amino acids mix + 0,2 µM TRAM
5 - WT MEF starved +100 nM insulin + 2.5 mM Glucose + 1x non-essential and essential amino acids mix + 1 µM AKT VIII
6 - WT MEF starved +100 nM insulin + 2.5 mM Glucose + 1x non-essential and essential amino acids mix + 1,5 µM AKT VIII
7 - Vim -/- MEF starved
8 - Vim -/- MEF starved +100 nM insulin + 2.5 mM Glucose + 1x non-essential and essential amino acids mix
9 - Vim -/- MEF starved +100 nM insulin + 2.5 mM Glucose + 1x non-essential and essential amino acids mix + 0,1 µM TRAM
10 - Vim -/- MEF starved +100 nM insulin + 2.5 mM Glucose + 1x non-essential and essential amino acids mix + 0,2 µM TRAM
11 - Vim -/- MEF starved +100 nM insulin + 2.5 mM Glucose + 1x non-essential and essential amino acids mix + 1 µM AKT VIII
12 - Vim -/- MEF starved +100 nM insulin + 2.5 mM Glucose + 1x non-essential and essential amino acids mix + 1,5 µM AKT VIII

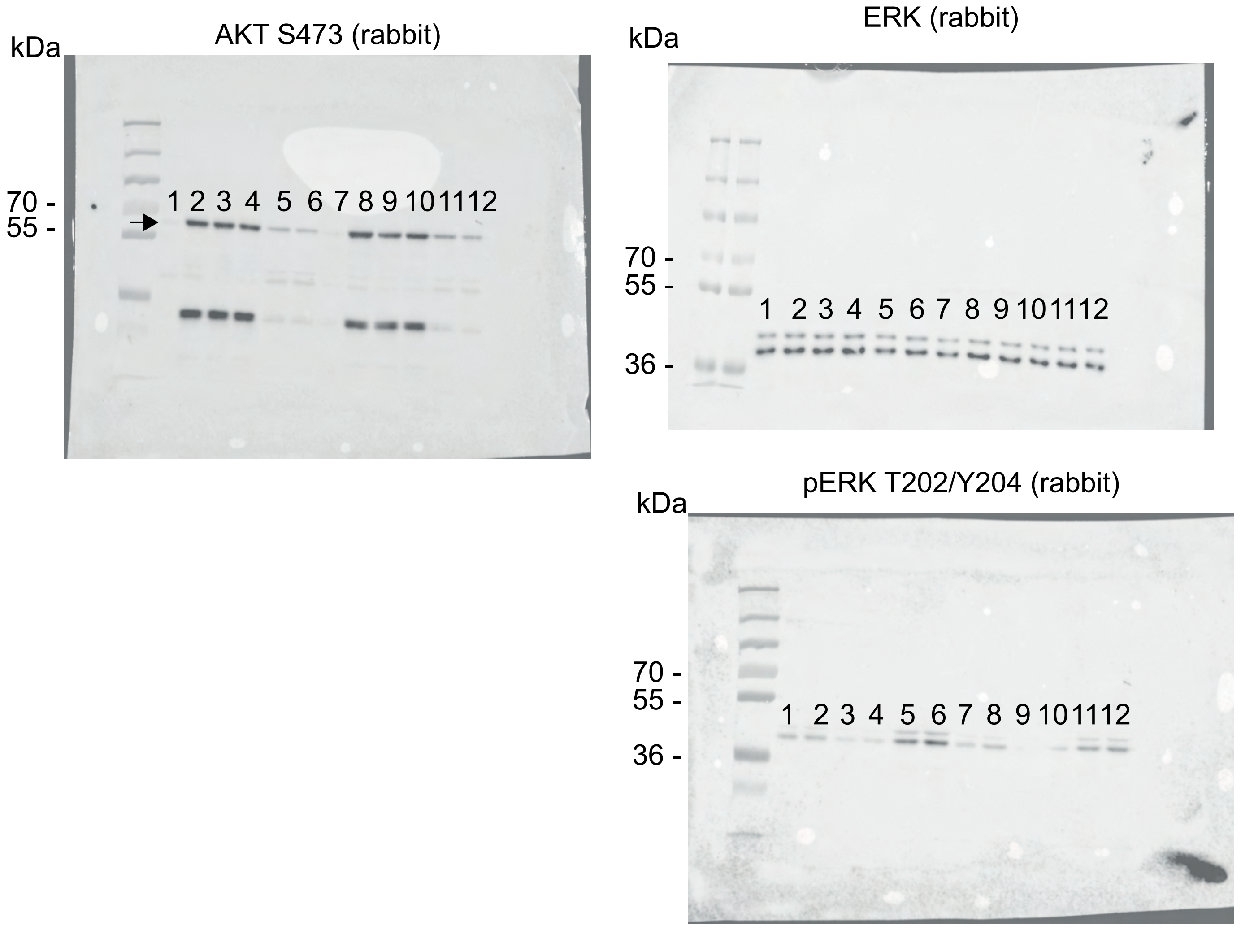

*when possible, images are an overlay of the chemiluminescence with the membrane, otherwise the chemiluminescence alone. Western blot are unedited and uncropped.

Fig.S2 c continued

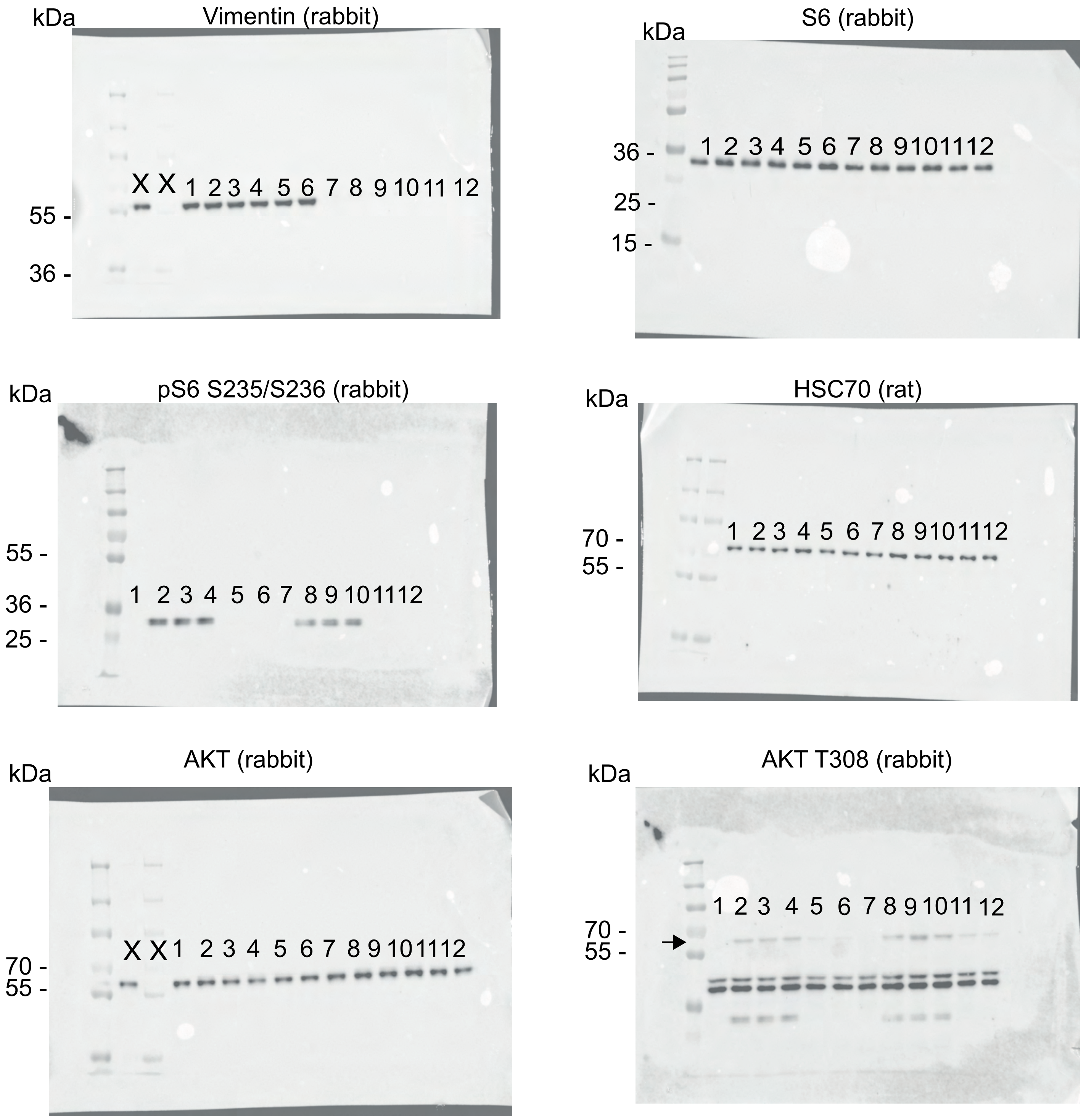

*when possible, images are an overlay of the chemiluminescence with the membrane, otherwise the chemiluminescence alone. Western blot are unedited and uncropped.

Fig.S5 b

Loading order:
1 - WT MEF in steaty state
2 - WT MEF in starvation RPMI with 2.5 mM Glucose
3 - WT MEF in starvation RPMI with 1x essential amino acids mix
4 - WT MEF in starvation RPMI with 2.5 mM Glucose and 1x essential amino acids mix
5 - WT MEF in starvation RPMI with 1x non-essential amino acids mix
6 - WT MEF in starvation RPMI with 2.5 mM Glucose and 1x non-essential amino acids mix
7 - Vim -/- MEF in steady state
8 - Vim -/- MEF in starvation RPMI with 2.5 mM Glucose
9 - Vim -/- MEF in starvation RPMI with 1x essential amino acids mix
10 - Vim -/- MEF in starvation RPMI with 2.5 mM Glucose and 1x essential amino acids mix
11 - Vim -/- MEF in starvation RPMI with 1x non-essential amino acids mix
12 - Vim -/- MEF in starvation RPMI with 2.5 mM Glucose and 1x non-essential amino acids mix

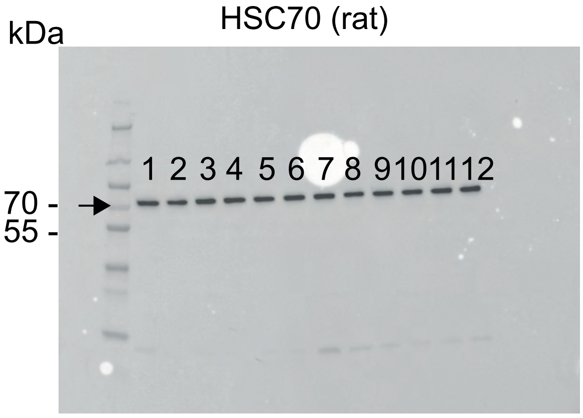

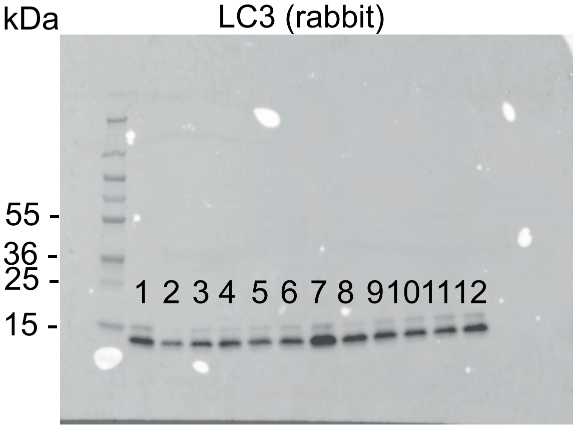

*when possible, images are an overlay of the chemiluminescence with the membrane, otherwise the chemiluminescence alone. Western blot are unedited and uncropped.
