# Supplementary material for: Cytoskeletal vimentin regulates cell size and autophagy through mTORC1 signaling
Source: PLoS Biol. 2022 Sep 13;20(9):e3001737. doi: 10.1371/journal.pbio.3001737 (PMC9469959; doi:10.1371/journal.pbio.3001737)
Supplement: S1 Table — (PDF) [file pbio.3001737.s010.pdf]

S1 Table. List of antibodies.

Application	Primary antibody	Product details	Secondary antibody	Product details
**Flow cytometry**	Vimentin Alexa Fluor® 647 Conjugate	CST (#9856)	-	-
	S6 Ser235/236	CST (#4856)	Rabbit 488	Thermo (#A11034)
**Immunostaining**	Vimentin	Biolegend (#919101)	Chicken 555	Thermo (#A21437)
	mTOR	CST (#2983)	Rabbit 488	Thermo (#A11034)
	LAMP1	SCBT (#19992)	Rat 633	Thermo (#A21247)
	HA-Tag (6E2)	CST (#2367)	Mouse 555	Thermo (# A28180)
	HA-Tag (C29F4)	CST (#3724)	Rabbit 488	Thermo (#A11034)
**Immunoblotting**	HSC70	Enzo (#ADI-SPA-815)	Rat HRP	GE (#NA935)
	LC3A/B	CST (#4108)	Rabbit HRP	Promega (#W401B)
	P70S6K	CST (#2708)		
	S6	CST (#2217)		
	4EBP1	CST (#9644)		
	P62	CST (#5114)		
	AKT	CST (#9272)		
	ERK	CST (#4695)		
	Insulin Receptor	CST (#3025)		
	P70S6K Thr389	CST (#97596)		
	S6 Ser235/236	CST (#4858)		
	4EBP1 Thr37/46	CST (#2855)		
	AKT Thr308	CST (#13038)		
	ERK Thr202/204	CST (#4370)		
	AKT Ser473	CST (#4060)		
	Insulin Receptor Tyr1150/1151	CST (#3024)		
	Vimentin	CST (#5741)		
	ULK1	CST (#8054)		
	ULK1 Ser757	CST (#14202)		
