# Supplementary material for: Cytoskeletal vimentin regulates cell size and autophagy through mTORC1 signaling
Source: PLoS Biol. 2022 Sep 13;20(9):e3001737. doi: 10.1371/journal.pbio.3001737 (PMC9469959; doi:10.1371/journal.pbio.3001737)
Supplement: Attachment — Submitted filename: Cover_Letter_and_Response_to_reviewers.pdf [file pbio.3001737.s011.pdf]

TURKU BIOSCIENCE

December 30, 2021

We are resubmitting our manuscript titled *"Cytoskeletal vimentin regulates cell size and autophagy through mTORC1 signaling"* by Ponnuswamy Mohanasundaram, Leila S Coelho Rato, Mayank Modi, Marta Urbanska, Franziska Lautenschläger, Fang Cheng, and John E. Eriksson to be considered for publication in *PLOS Biology*.

We thank you for the response to our previous submission PBIOLOGY-D-21-01157R1. Overall, we were delighted about the positive response to our study and we are grateful for the careful and thoughtful comments of the monitoring Editor and the reviewers. On the whole, the feedback we received has strengthened our observations and the conclusions that can be drawn from these observations. Moreover, importantly, the amendments and additional data provides us with a mechanism that we can present in the amended manuscript.

To summarize and recapitulate, we show that the cytoskeletal intermediate filament protein is required for normal mTORC1 signaling, a critical signaling determinant of cell size. Absence of vimentin in *Vim-/-* cells and mice results in anabolic deficiency due to disruption of vimentin-mediated mTORC1 activation. In this way, our study reveals a link between a major cytoskeletal complex and the key signaling system that determines both cell size and autophagy. Interestingly, our report also matches the phenotype described in the first report on a harmful mutation in the human gene encoding vimentin, which was shown to manifest itself as an anabolic deficiency in the form of lipodystrophy (Cogne et al. *Eur. J. Hum. Gen.* 2020; Eriksson *Eur. J. Hum. Gen.* 2020)[3,4].

Mechanistically, already the first submission demonstrated that vimentin controls mTORC1 mobility allowing access to lysosomes. In light of the major comments from our reviewers, we have especially aimed at strengthening this paradigm and to also unravel the underlying molecular mechanisms. To this end, we have introduced significant new data that shows that vimentin triggers mTORC1 activation by Rag mobilization and activation. Furthermore, we can now show that vimentin-links to autophagy through ULK1(8), the autophagy activating kinase that is required for to trigger autophagy by Beclin-1 phosphorylation. mTORC1 is known to inhibit autophagy by phosphorylating ULK1. The observed higher autophagy levels in the absence of vimentin, stem from reduced phosphorylation of ULK1 by mTORC1, which will result in ULK1-mediated activation of autophagy, as reflected by reduced levels of p62. We have also added new experiments to strengthen the data on autophagy and also redone the phenotype rescue experiments, as pointed out by the reviewers, now with highly convincing results, including also rescue of S6 phosphorylation. In addition, we have carefully modified the text according to the suggested amendments. Below is an itemized point-by-point reply to the comments of the editor and reviewers.

Our study reveals a new association of the cytoskeleton with cell size control, pointing towards a concerted bidirectional interplay between cell size signaling and structural regulation. Thus, we have confidence that this study should be of broad general interest for the readership of *PLOS Biology*. The results of our manuscript present original unpublished research and the manuscript is not being considered for publication elsewhere. All authors have read and approved the submitted version of the manuscript. The authors declare no conflict of interest.

As we believe that we have met all revision requirements, we hope that the manuscript would now be accepted for publication in *PLOS Biology*. We look forward to hearing from you.

Yours,

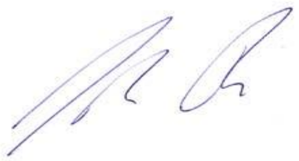

John Eriksson, Ph.D.
Professor of Cell Biology
Director General, Euro-Bioimaging ERIC

Correspondence to:
John E. Eriksson, Ph.D.
Professor of Cell Biology
Director General, Euro-Bioimaging ERIC
Turku Bioscience Centre
University of Turku & Åbo Akademi University
Tykistokatu 6B, FI-20520 Turku, Finland
Phone: int. +358-2-215 3313
Email: john.eriksson@abo.fi, john.eriksson@bioscience.fi
http://www.bioscience.fi/
https://www.eurobioimaging.eu/

References:

1. Cheng, F. *et al.* Vimentin coordinates fibroblast proliferation and keratinocyte differentiation in wound healing via TGF-β–Slug signaling. *Proceedings of the National Academy of Sciences* **113**, E4320–E4327 (2016).

2. Virtakoivu, R. *et al.* Vimentin-ERK Signaling Uncouples Slug Gene Regulatory Function. *Cancer Res.* **75**, 2349–2362 (2015).

3. Cogné, B. *et al.* A dominant vimentin variant causes a rare syndrome with premature aging. *Eur. J. Hum. Genet.* (2020).

4. Eriksson, J.E. Harmful vimentin manifests itself as multiorgan failure. *Eur. J. Hum. Genet.* **28**: 1139-1140 (2020).

5. Wang, R. C. *et al.* Akt-Mediated Regulation of Autophagy and Tumorigenesis Through Beclin 1 Phosphorylation. *Science* **338**, 956–959 (2012).

6. Kim, S., Wong, P. & Coulombe, P. A. A keratin cytoskeletal protein regulates protein synthesis and epithelial cell growth. *Nature* **441**, 362–365 (2006).

General observations:

As indicated above, we are delighted by the overall positive response to our study, especially by reviewer 1, and we thank all reviewers for their valuable comments many of which significantly strengthened the manuscript during the amendments. The most critical comments by reviewer 2 and 3 considered the lack of mechanistic details. However, we are convinced that our amended study provides both novelty and that our results reveal a new mechanistic level of mTORC1 regulation.

In its present form, the manuscript delineates the following mechanisms:

- The data clearly shows that vimentin promotes mTORC1 signaling by facilitating mTORC1 activation.
- The effects are at the level of localization of mTORC1 to lysosomes.
- Our data demonstrate that the suppression of autophagy is independent of growth factor signaling.
- Related to this, as the observed vimentin-dependent inhibition of autophagy is Akt-independent, it takes place by an alternative mechanism than the one proposed by Wang et al (Science 2013), in which vimentin and Beclin 1 are phosphorylated by AKT. We have introduced significant new data that shows that vimentin triggers mTORC1 activation by Rag mobilization and activation.
- We also have new data to show that vimentin- links to autophagy through ULK1(8), the autophagy activating kinase that is required for to trigger autophagy by Beclin-1 phosphorylation.
- Hence, we show that mTORC1 inhibits autophagy via ULK1 and independently of Akt. In this respect, related to autophagy, we are focusing on this part of the pathway, which is supported by unpublished interactomics data from our research team (which is extensive in its reach and presentation and beyond the scope of the current article).
- Our data demonstrate that the vimentin-mediated effects on p62 prevents autophagy from proceeding.
- Related to this result, importantly, vimentin engagement with p62 was shown to enhance cancer metastasis (Li et al, Carcinogenesis 2017; PMID: 28968743).
- In further support of the timeliness of our study, a recent study reported that vimentin regulates the volume of polyploidal giant cancer cells isolated from MDA-MB-231 cells (Xuan et al, PNAS, 2020; PMID: 33046658), highlighting the role of vimentin in cell size regulation in a completely different context and model system.
- We are also able to mechanistically demonstrate, by using constitutively active Rag B+C (as suggested by Reviewer 1), that vimentin determines Rag mobilization and activation and, thereby, mTOR signaling and localization.
- The autophagic flux can be difficult to assess from Western blots, for example, comparing LC3 I and LC3 II band can be misleading. To strengthen the autophagy data and to show autophagy flux more convincingly, we have now added data using the pMRX-IP-GFP-LC3-RFP-LC3ΔG plasmid (as published by Kaizuka et al. Mol Cell 2016; PMID: 27818143). During autophagy, GFPLC3 is cleaved and degraded in lysosome but RFP-LC3ΔG (it lacks cleavage site) is retained in the cells, which enables us to measure the autophagic flux.

Reviewer comments and point-by-point replies

Overall: We thank Reviewer 1 for the excellent comments. Addressing these items, significantly strengthened the manuscript.

Rev.1:

This is an interesting manuscript that identifies a new function/role for Vimentin in regulating activity of an important signaling pathway, the mTORC1 pathway, thereby affecting cell size. Overall, the results are interesting and the data are of good quality. The manuscript will definitely be of interest to a broad audience of people interested in cell size, nutrient sensing, insulin/mTORC1 signaling, growth, metabolism, and intermediate filaments.

I have two main issues which I think would be good to address prior to publication:

1. Fig 2f - the rescue of cell size by re-expression of Vim in the Vim KO cells does not look very good. Although there is a significant increase in size upon Vimentin re-expression, it only increases marginally compared to the size of the WT MEFs. This should be studied in more detail, because if true, it would suggest that most of the size reduction in the Vim- MEFs compard to WT MEFs is not due to lack of Vim (ie off-target). But most likely there is a technical explanation - e.g. too low expression of Vim in the pCMV-Vim transfected cells, or too high expression, or the transfection efficiency is too low and only hitting a small percentage of the cells. If the authors perform a western blot on WT MEFs, Vim- MEFs, and Vim- MEFs transfected with pCMV-Vimentin, how do the relative Vim protein levels look? Since this only shows the population average, if the Vim antibody also works for immunostaining, this can also be tested to see if perhaps only few cells are transfected but expressing very high levels, so that on average it looks like Vim expression is efficiently restored but in reality only a few cells have Vim expression. Possible work-arounds could be to stain Vim-KO + pCMV-Vim MEFs for Vim and then only measure the size of cells with good Vim expression, or to co-transfect GFP and only measure size of the green cells (which presumably will be co-transfected for GFP and Vim), or to use a CMV-Vim construct with a selectable marker and generate a stable cell line. In any case, it would be important to show in one graph the size of WT cells, Vim-KO cells and the rescued cells.

Response: In the rescue experiments, the seemingly small changes in cell size were due to low transfection efficiency, i.e., the presence of untransfected cells masked the real effect. To have a data set that better represents the reality, we have significantly improved the transfection efficiency (as demonstrated by Western blotting). The gained results give a better measure of the real vimentin-mediated effects on cell volume. Importantly, we have also added data to show that we were able to rescue the phosphorylation of S6 in Vim -/- MEFs by transfecting WT vimentin.

2. The data show that there is a defect in the ability of mTORC1 to relocalize to lysosomes in the Vim-/- cells upon nutrient/insulin restimulation. This can be due either to a defect in mTORC1 trafficking, or a defect in Rag function (i.e. they are not becoming active in order to bind mTORC1). Although I usually do not ask authors to do experiments during revisions that push the manuscript to expand beyond its initial findings, if the authors have time and interest, it would be relatively easy to transfect the Vim mutant MEFS with constructs that express constitutively active RagA+C (or WT RagA+C as a negative control - both available at Addgene I believe) to see if this rescues the TORC1 localization. If it does, then the defect is in

Rag activation. If it doesn't, then there's a defect in mTORC1 movement/transport. I think this would provide a nice additional step in the mechanistic understanding of how Vim affects mTORC1.

Response: We agree that this is a highly relevant aspect and suggestion to establish how vimentin determines mTORC1 signaling. We have now included new included experiments that show that constitutively active RagB+C rescue mTORC1 localization in Vim-/- cells. Hence, we can propose that vimentin regulates mTORC1 localization by determining Rag activation (Figure 5).

Minor Issues

1. It would be helpful in Fig. 3C to indicate the assay (incorporation of L-homopropargylglycine (HPG), or click-methionine) so that people don't need to look at the M&M to figure out what assay is being used.

Response: We have included the details of the protein synthesis assay in the figure legend.

2. From Fig. 4a-b, the authors conclude "some residual S6 activation could be observed… However, 4EBP1 was unvaryingly inhibited… These results imply that in Vim -/- cells there is a consistent inhibition of protein synthesis through hypophosphorylation of 4EBP1," This section is a bit confusing, and I believe the conclusions are not quite correct and should be rephrased. Firstly, since 4EBP inhibits translation, if 4EBP "is unvaryingly inhibited" then translation should be active. I think the authors don't mean that 4EBP is inhibited (ie phosphorylated by TORC1) since they then claim it is hypo-phosphorylated, but rather that 4EBP is constitutively activated in the KO MEFs. That said, I actually don't see a big difference between the reduction in TORC1 activity observable on the two downstream branches, S6K versus 4EBP. In the Vim- cells in Fig 4a, phosphorylation of both S6K and 4EBP is reduced compared to the WT cells, but still clearly visible in both cases. It looks like roughly a 50% reduction in both cases. In particular, looking at phospho-4EBP, I can clearly see a strong band in lanes 9-12. Also, if one looks at the band-shifting of total 4EBP, one clearly sees it is up-shifted in lanes 9-12. In fact, in Fig 4b, the phosho-4EBP signal as well as the up-shift in total 4EBP in the Vim- cells is very similar to that of the WT cells. So I think it is not correct to conclude that "there is a consistent inhibition of protein synthesis through hypophosphorylation of 4EBP1". I would conclude from Fig 4a-b that phosphorylation of both S6K and 4EBP are partially impaired, and likely both are contributing to the reduced translation rates in the KO cells.

Response: We have rephrased the critical sentences accordingly.

3. Fig 4C-D: the conclusion that "no increase in the colocalization could be observed in Vim -/- MEFs (Fig. 4c, d)." seems too strong to me. From the quantification in Fig 4d it looks like there is an increase in lysosomal TOR upon insulin stimulation also in the Vim- cells, but it's less strong than in WT cells. The fact that it's not statistically significant does not mean there is no effect (see PMID 30894741).

Response: We have rephrased these sentences accordingly.

4. The paragraph "S6 is phosphorylated at serine 234/235…" (page 5) would fit better before TORC1 localization is discussed because otherwise the storyline concludes there's an effect

on TORC1, then looks at the mechanism how this could be happening by looking at TORC1 localization, and then goes back to re-convince the reader that it's really TORC1 and not RSK (which it can't be anyways, because there's a clear effect also on S6K, which is a direct TORC1 target.)

Response: We have moved this paragraph before mTOR translocation.

5. ED Fig 3 is lacking a non-starved control, to draw the conclusion the authors draw that "nutrient starvation increases the conversion of LC3I to LC3II in both WT MEFs and Vim -/-".

Response: We have included non-starved control in LC3 blot (extended data figure 5b).

6. ED Fig 3 - I don't see the higher rate of conversion of LC3I to LC3II in the KO MEFs in this figure. If I compare the intensity of the lower band (LC3 II) in lanes 7-12, it looks lower than the intensity of the corresponding bands in lanes 1-6 ? Also, LC3 II should be used as a readout for autophagic flux only in combination with +/- Bafilomycin A, because otherwise the result is not interpretable (see PMID 28846632 section 3.1 - low LC3 II can either mean less autophagy or increased autophagic flux)

Response: We agree that this comment is highly relevant, as the autophagic flux can be difficult to assess from Western blots, for example, comparing LC3 I and LC3 II band can be misleading. Under normal conditions, LC3 II is low and if cells undergo autophagy LC3 II will indeed increase. Importantly, however, LC3II will start disappearing if cells undergo strong autophagy. To overcome these problems and to show autophagy flux more convincingly, we have now added data using the pMRX-IP-GFP-LC3-RFP-LC3ΔG plasmid (as published by Kaizuka et al. Mol Cell 2016; PMID: 27818143). During autophagy, GFPLC3 is cleaved and degraded in lysosome but RFP-LC3ΔG (it lacks cleavage site) is retained in the cells, in this way, we have shown autophagic flux by measuring the GFP and RFP fluorescent intensity ratio (Figure 6c, Extended Data Figure 5a). This provides strong evidence on a direct link between vimentin and autophagic flux.

7. Since autophagy activates mTORC1 (by providing amino acids) and mTORC1 inhibits autophagy (via phosphorylation of ULK1), the combination of low mTORC1 and high autophagy indicates most likely, as the authors conclude, that the causality relationship in the Vim-/- cells is that low mTORC1 causes low ULK1 phosphorylation and hence high autophagy. Nonetheless, the section on autophagy and how this relates to the mTORC1 phenotype is currently still a bit unresolved. A phospho-ULK1 western blot on the Vim- cells would clarify and provide the necessary evidence to support the mechanistic model shown in Fig. 6.

Response: Again, we agree that assessing the effects of vimentin on phospho-ULK1 helps us to understand how vimentin affect cell growth and autophagy through mTORC1 signaling. We show that vimentin is required for activation (phosphorylation) of ULK1 upon nutrient stimulation and is also important to maintain residual activity at nutrient depletion conditions (Figure 6a,d). These results suggest that vimentin inhibits autophagy by promoting mTORC1 signaling.

Rev.2:
The paper entitled "Cytoskeletal vimentin regulates cell size and autophagy through mTORC1 signaling" by Mohanasundaram et al. demonstrated their findings that vimentin controls mTORC1 mobility by allowing access to lysosomes. Though potentially interesting, this work lacks novelty and mechanistic insights. Additionally, their conclusions cannot be drawn based on the current results.

We thank Reviewer 2 for the positive outset and especially for the suggested link to Rag GTPase, which our added and amended data now shows to provide the missing link to vimentin.

As indicated above, we felt the criticism regarding lacking novelty and mechanistic details to be unwarranted as the manuscript provides significant advance and novelty in terms both providing a concrete link between the cytoskeleton and the major signaling pathway regulating cell size and delineating how this interaction takes place, as further outlined in the general response above and reiterated here:

- ***Our data clearly shows the novel role of vimentin in mTORC1 signaling.***
- ***Localization of mTORC1 to lysosomes prominently affected.***
- ***Our data also demonstrate that the vimentin inhibits autophagy independent of growth factor signaling.***
- ***Related to this, Wang et al (Science 2013) proposed that vimentin inhibit autophagy by binding to Beclin 1 and it is mediated by AKT***
- ***Here we are showing that vimentin inhibits autophagy via mTORC1 downstream target ULK1 and independently of Akt. We are focusing on this part of the pathway, as it is supported by unpublished interactomics data from our research team*** *(the interactomics data is extensive in its reach and presentation and beyond the scope of the current article)**.***
- ***In support of the timeliness of our study, a recent study reported that vimentin regulates the volume of polyploidal giant cancer cells isolated from MDA-MB-231 cells (Xuan et al, PNAS, 2020; PMID: 33046658), highlighting the role of vimentin in cell size regulation in a completely different model system.***
- ***-To add mechanistic details, we measured mTOR localization on lysosomes in WT and Vim-/- MEFs transfected with constitutively active RagB+C, which was suggested by reviewer1.***
- ***Our experiment results add new mechanistic details about vimentin role in mTORC1 signaling now delineating the molecular-level engagement of vimentin in the mTORC1 signaling pathway.***

Major points:
1. It is not unexpected that vimentin may play important roles in the regulation of cell size, since it is an intermediate filament protein. The results showed that vimentin knockout results in reduced mTOR localization at LAMP-1 positive vesicles. However, the authors did not explore the underlying mechanism. Does vimentin itself serve as tracks for lysosomal transportation of mTOR, or simply prevent its proteasomal degradation? Or does vimentin regulate mTOR transportation via rag GTPase? Now we are all left guessing. Additionally, the authors did not explain why mTORC2 activity was not affected by vimentin ablation. Although mTORC1 translocation and activation is impaired in Vim -/- MEFs, the authors cannot exclude that Vimentin may also regulate cell size via other signaling pathways such as Hippo, since rescue experiments were not performed.

Response: NB! Regarding the first comment, we have in fact addressed and ruled out the assumption that the cell size effect would be stemming from presence vs. absence of the intermediate filaments as a major cytoskeletal system. First of all, already the experiment in Fig.3a alone, showing that serum starvation reduced the size of WT MEFs to the same as Vim -/- MEFs (Fig 3a), is excluding this possibility. The WT would not decrease to the same size if the cell size effect would be a cytoskeletal effect, but instead this experiment clearly shows that the cell size reduction is a metabolic effect as it can be mimicked in WT cells by starvation. Furthermore, we observed the same effect in several different cell types at different states and cell cycle synchronization and, importantly, we specifically used primary bone marrow-derived dendritic cells to compare the results acquired from adherent fibroblasts with a non-adherent and spherical cell type. These cells possess a significantly less extensive cytoskeleton, including a relatively minute presence of vimentin in WT cells, which implies that possible structural effects from cytoskeletal elements are minimized. Furthermore, we used a microfluidic method to image the cells not only in the initial state, but also upon deformation in a narrow constriction of a microfluidic channel, where cytoskeletal structures are strained. The results from all these experiments emphasize the generic nature of the vimentin-dependent effect on cell size and also that this not a cytoskeletal effect per se, but one that is generated through a disturbance of the mTORC1 signaling pathway that we have identified in a causal link between vimentin and cell size regulation. All our other findings support this assumption. We have now more clearly stated the exclusion of a mere cytoskeletal effect in the text.

While we found that vimentin has a direct link to nutrient signaling, which is established to be integrated by mTORC1, we focused this study on how vimentin specifically regulates mTORC1 signaling. The link between vimentin and Hippo may be relevant for other aspects of vimentin-mediated signaling functions, but our unpublished data indicate that the link from vimentin to Hippo signaling it is not relevant for the observations related to cell size regulation, which our included results demonstrate to be limited to mTORC1 signaling.
The interplay between vimentin and Hippo signaling is of course a relevant and important question. Again, to reiterate the conclusion above, while we do have data on Hippo-coupled signaling, from what we have seen the data does not come across as relevant for this study and including this pathway in an already quite extensive study would be clearly beyond the scope of the current manuscript.

Regarding additional mechanistic details, we have included experiments suggested by reviewer 1, using constitutively active Rag B+C, which now provided the missing link between vimentin and mTOR localization.

We were somewhat surprised by the comment regarding mTORC2 activity, as mTORC1 has been established as the major nutrient sensor in the cell, whereas mTORC2 has not been coupled to regulation of cell size but rather being involved in cytoskeletal remodelling. While we do have data on mTORC2 signaling, at this point, this data comes across as irrelevant for this study and including this pathway in an already quite extensive study would lead us way beyond the scope of the current manuscript. Hence, we did not address this point since we wanted to focus specifically on mTORC1 as the major nutrient-mediated cell size signaling pathway.

2. To assess the autophagy flux in control or vimentin knockout cells, the most reliable marker, LC3B in the presence or absence of lysosomal protease inhibitors should be analyzed by Western blotting or Immunostaining.

Response: As outlined in the response to Reviewer 1, autophagic flux can be difficult to assess from Western blots, for example, comparing LC3 I and LC3 II band can be misleading. Under normal condition, LC3 II is low and if cells undergo autophagy LC3 II will indeed increase. Importantly, however, LC3II will start disappearing if cells undergo strong autophagy. To overcome these issues and to show autophagy flux more convincingly, we have used pMRX-IP-GFP-LC3-RFP-LC3ΔG plasmid (as published by Kaizuka et al. Mol Cell 2016; PMID: 27818143). During autophagy, GFPLC3 is cleaved and degraded in lysosome but RFP-LC3ΔG (it lacks cleavage site) is retained in the cells, in this way, we have shown autophagic flux by measuring the GFP and RFP fluorescent intensity ratio (Figure 6c). This additional data provides strong evidence on the effects on autophagic flux.

Minor points:
1. In all WB figures, the authors showed bands of HSC70 and did not explain why. I guess the authors used it as an internal control. However, it is unpredictable whether the level of HSC70 remains constant under stress conditions.

Response: HSC70 was used as a loading control, as we have found it to be among the most reliable loading controls showing consistent expression in different conditions (in contrast to other commonly used loading controls such as actin and GAPDH, which can be quite sensitive to different conditions). Under the used conditions, we have observed it not to be stress-responsive and, generally, HSC70 protein levels are well established, in contrast to the stress-responsive HSP70, to be relatively insensitive to stress. In parallel, we have observed equal loading by using tubulin as a loading control, which we have observed to be equallt reliable in terms of being insensitive to different conditions and treatments. The results obtained with tubulin show equally constant loading between different samples (data not shown).

2. The authors did not show detailed information of their Vimentin knockout mice. We're left speculating whether they are whole-body knockout or conditional knockout. Additionally, the nomenclature "WT" suggests the control mice were wild-type SVJ/129 mice, but not any floxed mice. Further, three pairs of WT and Vim-/- mice were not sufficient for statistical analysis.

Response: We apologize for not describing this more clearly. While we have used this mouse model extensively in our previous studies referred to in the methods of the manuscript (Fang et al. PNAS 2016; PMID: 27466403), we may have omitted some important details. The experiments are full knockout mice generated by heterozygote breeding. Heterozygous mice (129/Sv × C57BL/6) were used to generate vimentin-deficient homozygotes (VIM−/−) and WT offspring as described in the article above. We have included all these details in the relevant material and methods section.

Again, we apologize for the unclear description of our data. In fact, we used 6 pairs of mice, not 3 pairs of mice, and 4 pairs for the second age group which we felt was quite sufficient, since the results were so strikingly consistent. Furthermore, the mouse data was included to mainly show that lack of vimentin also has a physiological relevance at the level of body weight, organ size, and adipocytes cell size and we felt, in light of the highly consistent results and minute variation that this data would suffice. However, we could easily provide more data upon request.

Rev. 3: Han-Ming Shen - note that this reviewer has signed the review
The work by Mohanasundaram P et al aimed to examine the role of cytoskeletal protein vimentin in regulation of cell size-organ size and body weight through mTORC1 signaling. By using the vimentin KO mice and cells, provided both in vivo and in vitro evidence to support their main conclusion. However, the data provided were largely observational, lacking some essential mechanistic understanding on the regulatory role of vimentin on mTORC1 and cell/organ/body size.

We thank Reviewer 3 for his valuable comments and are delighted that about the overall positive assessment of our manuscript. Regarding the notion of about the results being largely observational, we feel, as indicated in the above sections, that our data is quite far beyond observational and, in fact, quite detailed as the manuscript provides significant advance and novelty in terms both providing a concrete link between the cytoskeleton and the major signaling pathway regulating cell size and also delineating how this interaction takes place.

These comments were already outlined above, but we reiterate our response here:

- ***The data clearly shows that vimentin promote mTORC1 signal either with through activator or an inhibitor of mTORC1.***
- ***The effects are clearly seen at the level of localization of mTORC1 and activation.***
- ***Our data demonstrate that the suppression of autophagy is independent of growth factor signaling.***
- ***Related to this, as the observed vimentin-dependent inhibition of autophagy is Akt-dependent, it takes place by an alternative mechanism than the one proposed by Wang et al (Science 2013), in which vimentin and Beclin 1 are phosphorylated by AKT.***
- ***Here we show that vimentin inhibits autophagy via ULK1 and independently of Akt. Hence, with respect to autophagy, we are focusing on this part of the pathway, which is supported by unpublished interactomics data from our research team.***
- ***In support of the timeliness of our study, a recent study reported that vimentin regulates the volume of polyploidal giant cancer cells isolated from MDA-MB-231 cells (Xuan et al, PNAS, 2020; PMID: 33046658), highlighting the role of vimentin in cell size regulation in a completely different model system.***

We have shown novel role vimentin in mTORC1 signaling, however due to lack of mechanistic details, we have included new experimental data which was suggested by reviewer 1, measuring mTORC1 localization on lysosome after transfecting constitutively active Rag B+C. We found that vimentin promote mTORC1 signaling by activating Rag GTPase which set forth the vimentin role in mTOR signaling and localization.

Major comments:
1. No sufficient evidence on autophagy. In this study, the authors only measured p62 protein level which is insufficient in making any conclusion on autophagy. The authors need to refer to the latest Autophagy guidelines (Klionsky DJ et al., Autophagy 2021) for measuring autophagic flux.

Response: As outlined in the response to Reviewer 1, autophagic flux can be difficult to assess from Western blots, for example, comparing LC3 I and LC3 II band can be misleading. Under normal condition, LC3 II is low and if cells undergo autophagy LC3 II will indeed increase. Importantly, however, LC3II will start disappearing if cells undergo

strong autophagy. To overcome these issues and to show autophagy flux more convincingly, we have used pMRX-IP-GFP-LC3-RFP-LC3ΔG plasmid (as published by Kaizuka et al. Mol Cell 2016; PMID: 27818143). During autophagy, GFPLC3 is cleaved and degraded in lysosome but RFP-LC3ΔG (it lacks cleavage site) is retained in the cells, in this way, we have shown autophagic flux by measuring the GFP and RFP fluorescent intensity ratio (Figure 6c). This data provides strong evidence to support our conclusions regarding the autophagic flux effects.

2. In this study, the authors did not address the point whether suppression of autophagy by vimentin (via activation of mTORC1) is involved in reduced cell size.

Response:

- ***The article indicates that vimentin does both, i.e. regulates both autophagy and is linked to mTORC1 signaling. To address this question in greater detail, we employed the phospho-ULK1 as a marker, which was also suggested by reviewer 1. This experiment determines to what extent vimentin regulates cell size through activation of mTOR or by suppression of autophagy.***
- ***Vim-/- showed lower level of phospho-ULK1 as compared to WT upon stimulation and in nutrient depletion conditions (Figure 6a, d). This suggest that vimentin inhibits autophagy by promoting mTORC1 signaling.***

3. Figure 2: The authors demonstrated restored cell size by reconstitution of vimentin in vimentin KO cells. Unfortunately they failed to conduct similar experiments for the subsequent experiments on mTOR activation, protein synthesis and autophagy, etc.

Response: We have performed vimentin rescue experiments and measured P-S6 level in vimentin positive cells through flow cytometry. Our results clearly showed that transfection of wild type vimentin in MEFs Vim-/- rescue pS6 level after stimulation. We also feel that the reviewer may agree with us that is not meaningful and relevant to reconstitute the full experimental panel with rescue experiments.

4. Figure 3C: The data on mTORC1 lysosomal localization is not convincing. Also, the figure legend did not provide clear info on labeling of mTORC and LAMP1.

Response: While the results are in line with and similar to several previous studies using mTORC1 localization to lysosomes as a measure of activation (Sancak et al. Cell 2010 PMID: 20381137; Betz et al. J. Cell Biol. 2013 PMID: 24385483, Orozco et al. Nat. Metabol. 2020 PMID: 32719541) we are able to provide more details and data on the lysosomal localization. We apologize if the labelling was unclear and we have included labels and information on all microscopy panels in order for the reviewers to interpret our results without difficulties.

5. Another missing mechanistic piece is how vimentin promotes mTORC1 lysosomal localization. Via direct interacting?

Response: As mentioned above, we have an extensive interactomics study (mass-spectrometry-based) in the works. In this study, we have identified a number of vimentin-interacting partners. Five to six proteins make mTORC1 complex and, apart from that, mTORC1 receives signals from different protein complexes. Validating which of those are bona-fide vimentin interacting partners will require a considerable amount of time and the

mentioned study is in terms of its breadth and volume clearly beyond the scope of the current manuscript, which we feel significantly advances the mechanistic insight with the included data.

6.Based on the model established by DM Sabatini, mTOR lysosomal localization mainly occurs to AA stimulation, but not to glucose or insulin, which is not consistent with the model presented in Figure 6.

Response: It is well established that amino acid stimulation is required for mTOR translocation to lysosomes. However, recently, several studies from the Sabatini lab showed that glucose also regulates mTOR signaling and translocation (PMID: 32719541, PMID: 23263183). Hence, the cartoon figure was designed to recapitulate this latest knowledge, indicating that amino acid and glucose regulate mTORC1 translocation and that insulin, in contrast, mainly promotes growth factor signaling.
