# Supplementary material for: Cytoskeletal vimentin regulates cell size and autophagy through mTORC1 signaling
Source: PLoS Biol. 2022 Sep 13;20(9):e3001737. doi: 10.1371/journal.pbio.3001737 (PMC9469959; doi:10.1371/journal.pbio.3001737)
Supplement: Attachment — Submitted filename: Response to reviewers.pdf [file pbio.3001737.s013.pdf]

Rev. 1:

The authors have nicely addressed the issues raised in the original review.

We thank reviewer 1 for the valuable input throughout the revision process that resulted in a significantly strengthened study and report.

Rev. 2:

The authors have added new data in the resubmitted paper entitled "Cytoskeletal vimentin regulates cell size and autophagy through mTORC1 signaling", to substantiate their findings. In general, the efforts made by the author to revise their manuscript, especially the mechanism by which vimentin regulates cell size and autophagy, is commendable. However, some issues should be addressed to help to strengthen the impact of this manuscript.

Major points:

1. The authors claimed that "Vim-/- cells showed lower level of phospho-ULK1 as compared to WT upon stimulation and in nutrient depletion conditions", in response to "the authors did not address the point whether suppression of autophagy by vimentin (via activation of mTORC1) is involved in reduced cell size", the 2nd major comment by reviewer 3. However, these data are still observational and do not help to establish a causal link between suppression of autophagy by vimentin and reduced cell size. To address this point, the authors should inhibit ULK activity in Vim-/- cells (e.g., ectopic expression of dominant-negative mutant of ULK) and measure the change in cell size.

We thank reviewer 2 for this valuable suggestion. We, indeed, recognised that this observation does not provide a direct causal link between vimentin and ULK1 in terms of autophagy regulation. Therefore, as suggested by reviewer 2, we have used a dominant-negative mutant ULK1 (https://www.addgene.org/22897/) to address this point. The requested experiment provided the answer to the question raised by reviewer 2 (/reviewer 3), the dnULK1 had no effect on cell size when introduced into Vim-/- MEFs, thereby providing the answer to the question of reviewer 2, as summarized in the part of the Result section that deals with ULK1-mediated regulation of autophagy.

2. In figure 5, a parallel western blotting figure is required to assess transfection efficiency of Rag-WT or CA plasmids. Besides, it is better to silence endogenous Rag GTPase before ectopic expression of Rag-WT or CA, which may render the results more significant and convincing.

We thank reviewer 2 for this comment. As our analysis was performed with imaging, we used an antibody against the HA-tag to detect transfected cells. Consequently, the data analysis was performed using only the HA-positive cells as an indication that each and every cell that was analysed was expressing our constructs. In this respect, we did not find Western blotting essential, as we only analysed the HA-positive cells that were expressing the plasmids. We recognise that we would perhaps get more striking results by silencing first the endogenous Rag. However, as the results obtained supported our hypothesis, even with endogenous Rag expressed, we thought it was not worth it to increase the experiment time and error sources by doing an additional silencing step that would add to the stress. This aspect is especially valid, as vimentin-deficient fibroblasts are subject to significant stress levels (because vimentin acts as a stress protector). Hence, additional silencing would be an unnecessary source of extra stress that could possibly affect cell signalling and, by extent, our conclusions.

3. The link between autophagy and cell size should be described briefly either in the introduction or the discussion section.

This is a great point has been corrected accordingly.

<u>Rev. 3:</u>

In this revised MS, the authors have made significant efforts in addressing the reviewers' comments. However, to this reviewer, there are several important issues that damps my enthusiasm to this work.

1. In the rebuttal (which is rather lengthy and redundant), the authors failed to provide the following to each of the points raised by the reviewers: (i) is there any new experiment done? (ii) if yes, what types of new experiments and where were the new data presented in the revised MS (either main figures or sup. figures, with figure # and panel #), and (iii) how the text in the MS was revised and where is the revision (page # and line #). Without such information, it is very hard for the reviewers to understand and to assess the revision and improvement made by the authors.

We thank reviewer 3 for recognising the significant improvements made to this manuscript. We also apologize for not being able fulfil the expectations when it comes to itemizing our amendments. However, we tried, in fact, to list all these amendments in the responses to the individual reviewers, which was the reason the response may have come across as somewhat redundant, as we made sure each and every reviewer got a full set of answers to their queries without having to consult responses to other reviewers. To address the shortcoming indicated by reviewer 3, we included now an itemized list of all changes and amendments made according to the procedure suggested by reviewer 3 (to be found at the end of the point-by-point reply).

2. Very disappointedly, the authors did not take the reviewers' comments on the proper measurement of autophagy flux seriously. For instance, the authors did not use the lysosomal inhibitors to examine the autophagic flux as clearly suggested by Reviewer 2. The new data added is good but not good enough to reach a solid conclusion on the enhanced autophagy level in Vim-/- cells.

Lysosomes are central hubs in regulating anabolic and catabolic processes through mTORC1 signalling. Blocking lysosomal function with lysosomal inhibitors not only blocks autophagy, but also affects mTORC1 signalling (PMID: 28846632, PMID: 22053050). The use of LC3 GFP/RFP is standardised way to measure the autophagic flux that is very well accepted in the field. LC3 GFP is degraded during autophagy, whereas LC3 RFP delta G is not, serving as an internal control (https://doi.org/10.1016/j.molcel.2016.09.037). Thus, in fact, we were conviced that using those constructs would be the best way to conclude the autophagy levels in vim -/- cells. In Figure 6a (and respective supplementary data), the ratio difference is quite striking regardless of the condition. Therefore, we believe that this experiment shows clearly that vimentin-deficient fibroblasts have higher autophagy levels.

3. The mTORC1 lysosomal localization data presented in Figure 4c and 5a were somehow not consistent and not convincing. For instance, in Figure 4c-top left panel (WT-Starved cells) Lamp1 dots were mainly in the nuclei. In Figure 5a, the two panels at the right, the fluorescence intensity of Lamp1 was much weaker than the two panels at the left. Such inconsistence thus raised significant technical Qs for the validity of the methods used in this study.

Important to observe that the figure represent experiments that were performed under completely different experimental conditions. Hence, the differing results do not reflect

inconsistencies but, in fact, differences in outcome depending on the different stimuli and experimental conditions that have been used in the given experiments. In Figure 4C, we measured mTORC1 and lysosome localization under starvation and stimulation conditions. In Figure 5A, we measured mTORC1 and lysosome localization in Rag GTPase WT and constitutively active transfected cells under steady-state conditions. Nutrient availability is well-established to modulate lysosome positioning. Under starvation, lysosomes colocalize around the nucleus, whereas stimulation causes lysosomes to localize close to upstream signalling modules (PMID: 21394080). In Figure 4C, lysosome localization towards nuclei is prominently visible in WT starved cells because its plasma membrane spreads out widely, and cells look bigger than Vim-/-. In Figure 5A, we measured mTOR and lysosome localization in Rag GTPase WT and Rag GTPase constitutively active (CA) transfected cells. Rag GTPase CA readily translocates into the lysosome without any stimulation. However, Rag GTPase WT requires nutrients for its activation and translocation. Constitutively active Rag GTPase recruits mTOR to the lysosome membrane more efficiently than Rag GTPase WT, which could increase mTOR signal (also, green colour looks brighter to the human eye than other colours) and masks Lamp1 signal in Rag GTPase CA. In supplementary Extended Data Figure 4, we have included grey scale images of all the channels, in which we do not find a big difference in fluorescent intensity between mTOR and Lamp1.

The colocalization experiment was performed with standard protocols and we used the same settings for all samples. Moreover, our calculations were made based on the raw data, whereas the representative images are adjusted for brightness and contrast. We used Mander's co-efficient to calculate colocalization to overcome intensity differences between the two channels. Mander’s co-efficient calculates colocalization based on the co-occurrence of two fluorophores, and it does not use fluorescent intensity to calculate colocalization. Moreover, to avoid signal crosstalk between two channels, we stained mTOR with green fluorophore Alexa flour 488 and Lamp1 stained with far-red fluorophore Alex flour 633.

Notably, all these results are consistent with the postulated paradigm.

4. Throughout the whole study, the effect of glucose on mTORC1 and AKT activation and autophagy is actually very marginal and minor (Figure 4a, 4b, Figure 6a, 6b, 6d). Therefore, it is not appropriate to include glucose in the illustration in Figure 7.

Amino acids and glucose were included in the model to give a general picture of Rag GTPases in mTORC1 signaling, as the general understanding in the field is that amino acids and glucose regulate mTORC1 signalling through Rag GTPases. However, we agree that the effects of glucose on mTORC1, AKT activation, and autophagy are minimal. Our graphical model intends to primarily describe that vimentin regulates mTORC1 signalling through Rag GTPases. The intention was not specify that vimentin regulates this process through amino acids and glucose or amino acids alone. As we agree with the notion of glucose not having a major influence in the context of our studies we are happy amend the the illustratation accordingly. The amended Fig. 7 reflects this change.

List of amendments and changes

1. As requested by Reviewer 2 (major point 1), we measured cell size in Vim-/- MEFs transfected with dominant-negative ULK1. Results in Extended Data Figure 6 and results described on page 6, line 208.

2. As requested by Reviewer 2 (major points 3), we have now included text about the link between cell size and autophagy in the introduction, on page 3, line 15.

3. Based on the Editor and Reviewer 3 (comments #4), we have made changes in our graphical model Figure 7.

4. Minor amendments to the text and references have been made.

5. All amendments in the manuscript are visible by tracked changes.
